# Prostate-Specific Membrane Antigen Targeted Deep Tumor Penetration of Polymer Nanocarriers

**DOI:** 10.1021/acsami.2c15095

**Published:** 2022-11-01

**Authors:** Niranjan Meher, Gary W. Ashley, Anil P. Bidkar, Suchi Dhrona, Cyril Fong, Shaun D. Fontaine, Denis R. Beckford Vera, David M. Wilson, Youngho Seo, Daniel V. Santi, Henry F. VanBrocklin, Robert R. Flavell

**Affiliations:** † Department of Radiology and Biomedical Imaging, University of California, San Francisco, California 94143, United States; ‡ ProLynx Inc., San Francisco, California 94158, United States; § Helen Diller Family Comprehensive Cancer Center, University of California San Francisco, San Francisco, California 94143-0981, United States; ∥ Department of Pharmaceutical Chemistry, University of California, San Francisco, California 94158-2517, United States

**Keywords:** positron emission tomography (PET) imaging, prostate-specific membrane antigen (PSMA), polymer nanocarriers, deep tumor penetration, enhanced permeability and retention (EPR) effect

## Abstract

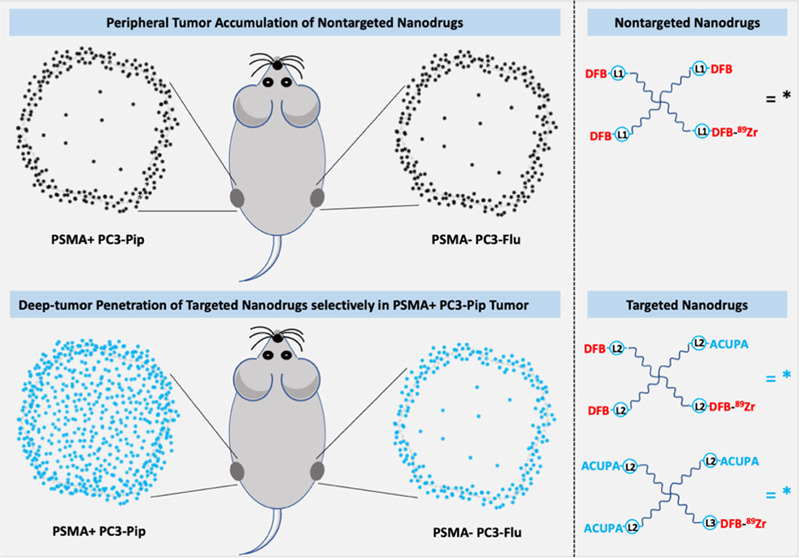

Tumoral uptake of large-size nanoparticles is mediated by the enhanced permeability and retention (EPR) effect, with variable accumulation and heterogenous tumor tissue penetration depending on the tumor phenotype. The performance of nanocarriers *via* specific targeting has the potential to improve imaging contrast and therapeutic efficacy *in vivo* with increased deep tissue penetration. To address this hypothesis, we designed and synthesized prostate cancer-targeting starPEG nanocarriers (40 kDa, 15 nm), [^89^Zr]PEG-(DFB)_3_(ACUPA)_1_ and [^89^Zr]PEG-(DFB)_1_(ACUPA)_3_, with one or three prostate-specific membrane antigen (PSMA)-targeting ACUPA ligands. The *in vitro* PSMA binding affinity and *in vivo* pharmacokinetics of the targeted nanocarriers were compared with a nontargeted starPEG, [^89^Zr]PEG-(DFB)_4_, in PSMA+ PC3-Pip and PSMA– PC3-Flu cells, and xenografts. Increasing the number of ACUPA ligands improved the *in vitro* binding affinity of PEG-derived polymers to PC3-Pip cells. While both PSMA-targeted nanocarriers significantly improved tissue penetration in PC3-Pip tumors, the multivalent [^89^Zr]PEG-(DFB)_1_(ACUPA)_3_ showed a remarkably higher PC3-Pip/blood ratio and background clearance. In contrast, the nontargeted [^89^Zr]PEG-(DFB)_4_ showed low EPR-mediated accumulation with poor tumor tissue penetration. Overall, ACUPA conjugated targeted starPEGs significantly improve tumor retention with deep tumor tissue penetration in low EPR PC3-Pip xenografts. These data suggest that PSMA targeting with multivalent ACUPA ligands may be a generally applicable strategy to increase nanocarrier delivery to prostate cancer. These targeted multivalent nanocarriers with high tumor binding and low healthy tissue retention could be employed in imaging and therapeutic applications.

## 1 Introduction

In the United States, prostate cancer is the most prevalent noncutaneous cancer in men and the second most common cause of cancer deaths.^1,2^ Glutamate carboxypeptidase II, commonly known as prostate-specific membrane antigen (PSMA), is an overexpressed cell surface enzyme on prostate cancer cells and a valuable clinical biomarker of prostate cancer.^3^ The recognition site is composed of two pockets with zinc ions, the nonpharmacophore pocket (S1) and the glutamate-sensing pocket (S1′). A urea-based ligand (ACUPA) with free carboxylic acid groups interacts with the S1′ pocket of PSMA selectively and has been employed in several single-photon emission computed tomography (SPECT) and positron emission tomography (PET) imaging and theranostic agents for prostate cancer.^3−6^^177^Lu-based PSMA-targeted radiotherapy is currently utilized in the clinic to treat metastatic prostate cancer.^7−9^ Inspired by promising clinical studies, there has been increased interest in utilizing PSMA targeting for nanoparticle delivery for imaging and therapy.^10−17^

In parallel with these developments, nanoparticle drugs have found increasing use in medicine.^18−22^ However, the effectiveness of these drugs is not uniform, which may be due in part to incomplete tumor penetration of the large-size macromolecules.^23^ Large-size nanoparticles may have nonspecific tumor accumulation because of the enhanced permeability and retention (EPR) effect.^24−26^ The EPR effect is the mechanism by which large-size macromolecules or nanoparticles of >10 nm diameter nontargeted drugs get accumulated in tissues with defective vasculature and impaired lymphatic drainage.^19,27,28^ EPR-mediated accumulation of nanoparticles in tumors with abnormal vascular architecture has been well established and a widely accepted strategy for the effective delivery of nanoparticles to tumors.^19,29^ However, the magnitude of the EPR effect is governed by various variables, including nanoparticle size, *in vivo* pharmacokinetics, vasculature, tumoral microenvironment, and the presence of macrophages.^27,30,31^ Thus, depending on the tumor phenotype, nanoparticles often lack deep tumor tissue penetration, limiting drug delivery and therapeutic efficacy.^24,30^ Furthermore, relatively large-size nanoparticles with strong target binding affinity may suffer from the binding site barrier (BSB) effect, in which the nanoparticles bind to cells peripheral to blood vessels, blocking their further diffusion into the bulk tumors.^13,23,32,33^ Overall, the nanoparticle size and the tumor phenotype play roles in both passive and active tumor uptake, making it challenging to design target-specific nanoparticles with longer retention times and tumor penetration.

**Figure 1:**
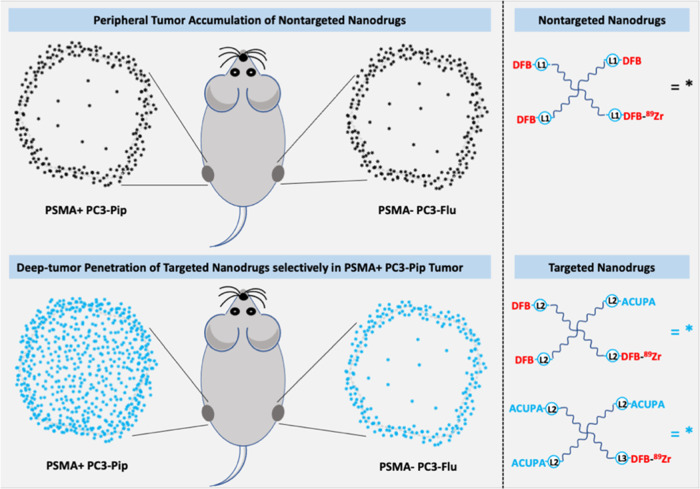
Graphical representation of PSMA-targeted polymer nanocarriers tagged with ^89^Zr radioisotope, demonstrating significantly improved deep tumor penetration in PSMA+ prostate cancer xenograft.

Previously, we have explored 4-armed starPEG nanocarriers of 40 kDa conjugated with ^89^Zr chelator deferoxamine B (DFB) as nontargeted PET radiopharmaceuticals in MX-1 and HT-29 tumor models. These studies demonstrated EPR-mediated high tumor accumulation and retention (about 10%ID) even after 9 days postinjection.^25,34,35^ In contrast, PET imaging studies of radiolabeled macromolecules and nanoparticles in preclinical human prostate cancer tumor models like CWR22rv1, DU-145, and PC3 demonstrated an “EPR low” effect with reduced tumor accumulation and retention.^13,30,31^ Thus, we hypothesized that conjugating the PSMA-targeted ACUPA ligands to large-size nanocarriers would potentially improve tumor uptake with enhanced retention and tumor tissue penetration, essential and desired characteristics for therapeutic efficacy. In this study, we evaluated three 4-armed starPEG_40kDa_ nanocarriers with zero, one, or three copies of PSMA-targeted ACUPA ligands and compared their *in vitro* PSMA binding affinity in PSMA+ PC3-Pip and PSMA– PC3-Flu cell lines. The *in vivo*-targeted uptake and deep tumor uptake of those starPEGs were demonstrated using PET imaging and organ biodistribution studies in a mouse model bearing dual prostate cancer xenografts of PSMA– PC3-Flu and PSMA+ PC3-Pip (Figure 1).

## 2 Methods

### 2.1 Materials and Instrumentations

The 4-armed PEG_40kDa_-(NH_2_)_4_ was purchased from SINOPEG (Fujian, China). ^89^Zr-oxalate was procured from three-dimensional (3D) Imaging (Little Rock, AR), p-SCN-Bn-deferoxamine from Macrocyclics (Plano, TX), and deferoxamine mesylate from Sigma-Aldrich (Rockville, MD). RPMI-1640 media, penicillin–streptomycin (P/S) solutions, and fetal bovine serum (FBS) were purchased from Life Technologies (Carlsbad, CA) and Thermo Fisher Scientific (Waltham, MA). Other chemicals (solvents, reagents, and building blocks) were bought from Thermo Fisher Scientific, VWR, or Sigma-Aldrich and used without further processing. ^1^H and ^13^C NMR spectra were recorded on Bruker 400 and 100 MHz NMR spectrometers, respectively. Chemical shifts were shown in parts per million (ppm, δ). High-resolution mass spectrometry (HRMS) was recorded at QB3/Chemistry Mass Spectrometry Facility, University of California, Berkeley.

### 2.2 Synthesis of starPEG Conjugates

#### 2.2.1 General

High-performance liquid chromatography (HPLC) was performed using a 4.6 × 150 mm^2^ 5 μm 300 A Phenomenex Jupiter C18 reversed-phase column with a 15 min linear gradient of 0–100% acetonitrile/water/0.1% TFA (1.0 mL/min) beginning 2 min after injection, unless otherwise mentioned.

#### 2.2.2 Azido-ACUPA-tBu (**1**)

A solution of 6-azidohexyl succinimidyl carbonate (150 mg, 0.52 mmol, 1 equiv) in 1 mL of DCM was mixed to ACUPA-tBu (283 mg, 0.58 mmol, 1.1 equiv) in 5 mL of DCM containing 176 μL of DIPEA. The reaction was subjected to magnetic stirring at room temperature for 24 h under argon. After completion, the reaction mixture was evaporated under reduced pressure. The mixture was eluted with 5% methanol in dichloromethane in a silica gel packed column chromatogram to produce pure Azido-ACUPA-tBu (**1**) (282 mg, 74% yield). ^1^H NMR (400 MHz, CDCl_3_): d 5.17 (br, 2H), 4.96 (br, 1H), 4.33 (m, 2H), 4.04 (m, 2H), 3.26 (t, 2H), 3.14 (m, 2H), 2.30 (m, 2H), 2.07 (m, 1H), 1.85 (m, 1H), 1.74 (m, 2H), 1.60 (m, 6H), 1.45–1.39 (m, 33H). ^13^C NMR (100 MHz, CDCl_3_): d 172.56, 172.44, 157.04, 156.99, 82.22, 81.87, 80.66, 64,74, 53.42, 53.12, 51.49, 40.64, 32.79, 31.72, 29.83, 29.53, 29.05, 28.89, 28.53, 28.21, 28.15, 26.54, 25.62, 22.35. HRMS for C_31_H_57_N_6_O_9_ [M + H]^+^ 657.4187 (calcd: 657.4109).

#### 2.2.3 Azido-ACUPA (**2**)

Azido-ACUPA-tBu (**1**) (450 mg, 0.68 mmol) was dissolved in 3 mL of anhydrous DCM, and 3 mL of trifluoroacetic acid (TFA) was added to it. The mixture was stirred for 16 h at room temperature. On completion, the reaction mixture was evaporated at reduced pressure. The residue was dissolved in 5 mL of H_2_O, and the solvent was evaporated at reduced pressure again to remove any remainings of TFA. The residue was dissolved in CHCl_3_ and evaporated to yield the product Azido-ACUPA (**2**). ^1^H NMR (400 MHz, CDCl_3_): d 4.31 (m, 1H), 4.24 (m, 1H), 4.02 (m, 2H), 3.88 (t, 1H), 3.57 (s, 1H), 3.21 (q, 2H), 3.09 (t, 2H), 2.56 (m, 1H), 2.51 (s, 1H), 2.41 (m, 2H), 2.14 (m, 1H), 1.81 (m, 3H), 1.63 (m, 4H), 1.47–1.35 (m, 10H). ^13^C NMR (100 MHz, CDCl_3_): d 176.45, 176.40, 175.82, 160.14, 159.29, 65.66, 58.66, 55.84, 53.50, 52.36, 47.92, 33.20, 31.08, 30.49, 29.83, 28.94, 27.47, 23.84, 18.70. HRMS for C_19_H_31_N_6_O_9_ [M – H]^−^ 487.2162 (calcd: 487.2231).

#### 2.2.4 Azido-DFB (**3**)

Deferoxamine mesylate (Sigma, 165 mg, 0.25 mmol, 1.0 equiv) was dissolved in 1 mL of water in a 15 mL Falcon tube and neutralized by 0.5 mL of 1 M Na_2_CO_3_. A solution of 6-azidohexyl succinimidyl carbonate (75 mg, 0.26 mmol, 1.04 equiv) in 1 mL of acetonitrile was mixed, leading to slow formation of a precipitate.^36^ The precipitate was collected by centrifugation after keeping 3 hours at room temperature, washed successively with water and acetonitrile, and dried under vacuum to afford the product (yield 60%, 110 mg). HPLC: *R*_v_ = 10.2 mL; purity 96% (evaporative light scattering detector (ELSD) detection). ^1^H NMR (400 MHz, DMSO-*d*_6_): d 9.63 (br, 1H), 9.58 (br, 2H), 7.76 (t, 2H), 7.02 (t, 1H), 3.91 (t, 2H), 3.45 (m, 6H), 3.31 (m, 4H), 2.99 (m, 4H), 2.94 (m, 2H), 2.57 (t, 4H), 2.27 (t, 4H), 1.50 (m, 10H), 1.41–1.30 (m, 11H), 1.21 (m, 6H). ^13^C NMR (100 MHz, DMSO-*d*_6_): d 171.95, 171.26, 170.11, 156.30, 63.38, 50.53, 47.08, 46.77, 38.40, 29.89, 29.09, 28.80, 28.56, 28.13, 27.55, 26.02, 25.80, 24.92, 23.48, 23.33, 20.32. HRMS for C_32_H_60_N_9_O_10_^+^ [M + H]^+^ 730.4465 (calcd: 730.4385).

#### 2.2.5 Synthesis of PEG-(DFB)_3_(ACUPA)_1_ (**4**)

A 100 mg/mL solution of PEG-(5HCyO)_3_(NH_2_)_1_ in DMF (1.0 mL, 100 mg, 2.5 mmol, 1.0 equiv) was mixed with a 45 mg/mL solution of Azido-DFB (**3**) in DMSO (9 mg, 12.3 mmol, 1.5 equiv) and kept at 37 °C for 48 h.^25^ The mixture was dialyzed against water (SpectraPor 2 membrane, 12–14 kDa cutoff), followed by methanol to remove unconjugated materials and then dried under reduced pressure to give PEG-(DFB)_3_(NH_2_)_1_ (**S1**). The residue was dissolved in 1 mL of acetonitrile, and the insoluble material was removed by filtration. The solution was mixed with 75 mL of a 10 mg/mL solution of 5-hydroxycyclooctyne (5HCyO) succinimidyl carbonate in acetonitrile (0.75 mg, 2.8 mmol, 1.5 equiv) and DIPEA (1 mL, 5 mmol, 2.8 equiv) for 30 min at ambient temperature.^37^ The mixture was added slowly to 10 mL of MTBE, and the precipitated product was collected, washed with MTBE, and dried to give PEG-(DFB)_3_(5HCyO)_1_ (**S2**), which was advanced without further characterization or purification.

PEG-(DFB)_3_(5HCyO)_1_ (**S2**) was dissolved in 1 mL of 0.1 M sodium phosphate (pH 7.4) and treated with a 25 mM solution of Azido-ACUPA (**2**) in 0.1 M sodium phosphate, pH 7.4, (200 mL, 5 mmol, 2.8 equiv) for 48 h at 37 °C and then dialyzed (SpectraPor 2 membrane, 12–14 kDa cutoff) against water, followed by methanol, and dried under reduced pressure to afford the product PEG-(DFB)_3_(ACUPA)_1_ (**4**) (yield 45%, 48 mg). ^1^H NMR is provided in the Supporting Information.

#### 2.2.6 Synthesis of PEG-(DFB)_1_(ACUPA)_3_ (**5**)

A 100 mg/mL solution of PEG-(5HCyO)_3_(NH_2_)_1_ in DMF (1.0 mL, 100 mg, 2.5 mmol, 1.0 equiv) was mixed with a 10 mg/mL solution of *p*-isothiocyanatobenzyl-desferrioxamine B (ITCBz-DFB) in DMSO (Macrocyclics, 2.8 mg, 3.7 mmol, 1.5 equiv) and kept at room temperature for 16 h. The mixture was dialyzed against water (SpectraPor 2 membrane, 12–14 kDa cutoff) to remove unconjugated materials and then dried under reduced pressure. The residue was dissolved in 0.1 M sodium phosphate, pH 7.0, to provide a 75 mg/mL solution of PEG-(5HCyO)_3_(DFB)_1_ (**S3**), which was advanced to the next step without further characterization or purification.

A mixture of 75 mg/mL PEG-(5HCyO)_3_(DFB)_1_ (**S3**) in 0.1 M NaP*_i_*, pH 7.0, (850 mL, 1.6 mmol, 4.8 mmol 5HCyO) and a 30 mM solution of Azido-ACUPA (**2**) in 0.1 M NaP*_i_*, pH 7.4, (400 mL, 12 mmol, 2.5 equiv) was kept for 48 h at 37 °C, resulting a single new product peak by HPLC. The mixture was dialyzed (SpectraPor 2 membrane, 12–14 kDa cutoff) against water followed by methanol and dried under reduced pressure to provide the product PEG-(DFB)_1_(ACUPA)_3_ (**5**) (yield 78%, 50 mg). ^1^H NMR is provided in the Supporting Information.

### 2.3 Cell Culture

Unless and otherwise specified, the PSMA– PC3-Flu and PSMA+ PC3-Pip cell lines were cultured in RPMI-1640 medium containing 10% FBS and 1% penicillin/streptomycin (P/S) at 37 °C with 5% CO_2_. The cells were obtained from Dr. Martin Pomper′s lab, Johns Hopkins University. According to experimental protocols, cells were trypsinized (0.25%) for 2–3 min to detach from the culture flasks for further passage or to seed the cells in suitable multiwell plates to perform cell-binding assays.

### 2.4 Competition Radioligand Binding Assay

Similar to our prior reported protocol, ^68^Ga-PSMA-11 was produced in a ^68^Ge/^68^Ga generator and used in a competition radioligand binding assay to acquire the IC_50_ values for the nanocarriers.^10,38^ Briefly, around ∼0.185 MBq (2.5 ng) of ^68^Ga-PSMA-11 along with different concentrations (0.01–100,000 nM) of the nonradiolabeled nanocarriers was treated to each well of 96-well plates containing PSMA+ PC3-Pip cells (∼20 k cells/wells). After 1 h incubated at room temperature, the radioactive medium was removed, and the cells were washed with PBS twice. The cells were lyzed with sodium hydroxide, and the radioactivity of the lysate in each well was counted in a Hidex γ counter. IC_50_ was determined by nonlinear regression analysis in Prism software (GraphPad).

### 2.5 ^89^Zr Radiolabeling of starPEGs

^89^Zr-oxalate (9 μL, 192.4 MBq) was neutralized with 9 μL of Na_2_CO_3_ (1 M), and 400 μL of NH_4_OAc (1 M) was added to the mixture. To this mixture, ∼4 mg of starPEG conjugates in 100 μL of deionized (DI) water was added and incubated for 1 h at 25 °C. The radiolabeled product was purified using a PD-10 size-exclusion desalting column (Fisher Scientific, Hampton, NH) and eluting with saline solution. Instant thin-layer chromatography (iTLC) was performed using silica gel-impregnated glass microfiber chromatography paper (Neta Scientific, Hainesport, NJ) and developed with 50 mM EDTA solution to confirm radiolabeling purity. The isolated-bound activities were 159.1–185 MBq. Multiple radiolabeling studies were carried out with different amounts of nanocarriers and ^89^Zr and are summarized in Table S1. The radiolabeling yields ranged from 26.27–46.25 MBq/mg.

### 2.6 *In Vitro* Saturation Binding Assay

PC3-pip cells were seeded in 12-well plates (∼100 k cells/well) 24 h prior to testing. Cells were washed with PBS twice, and each well was treated with 1 mL of growth media without/with 10 μM PSMA-2 (a previously described PSMA inhibitor)^37−39^ and incubated at 37 °C for 1 h. Then, different concentrations (1–1000 nM) of the ^89^Zr-radiolabeled starPEG nanocarriers were treated to the cells and incubated at 37 °C. After 1 h of incubation, the radioactive medium was removed, and cells were washed with PBS. The cells were lyzed with sodium hydroxide, and the radioactivity in each well was analyzed in a Hidex γ counter. The respective nonspecific-bound activities were substracted, and the dissociation constant (*K*_d_) was calculated by nonlinear regression on site-specific binding in Prism Software (GraphPad). These data were further used to show the PSMA binding affinity and blocking of the starPEG nanocarriers at 1 h.

### 2.7 *In Vitro* Binding and Blocking Assay

PC3-Flu and PC3-pip cells were seeded in 24-well plates (∼50 k cells/well) 24 h prior to testing. Cells were washed with PBS twice, and each well was treated with 0.5 mL of growth media with/without 10 μM PSMA-2 (a previously described PSMA inhibitor)^37−39^ and incubated for 1 h at 37 °C. Then, different concentrations (10–100 nM) of the ^89^Zr-radiolabeled starPEG nanocarriers were treated to the cells and incubated further at 37 °C. The radioactive medium was discarded at 4 or 24 h time points, and cells were lyzed with NaOH (5 N, 250 μL) after washing with PBS. The lysate was analyzed in a Hidex γ counter (along with the standard treated activity to calculate the % bound activity).

### 2.8 *In Vitro* Membrane-Bound and Internalization Assay

Four sets of the PC3-pip and PC3-flu cells were seeded in 24-well plates (∼50 k cells/well) for 24 h before assay. Each well was treated with 1 μM ^89^Zr-radiolabeled starPEG nanocarriers and incubated at 37 °C. At each time point (1, 2, 4, and 24 h), one set of PC3-flu and PC3-pip cells was washed with PBS twice and incubated with a mixture of 0.5 mL of ice-cold glycine (50 mM) and NaCl (150 mM) for 5 min at 4 °C. The acid buffer corresponding to the membrane-bound activity was collected. Then, the cells were lyzed with sodium hydroxide (5 N), and the lysate corresponding to the internalized activity was collected. The respective radioactivities were analyzed in a Hidex γ counter (Turku, Finland) along with the standard treated activity of the ^89^Zr-radiolabeled starPEG nanocarriers to calculate the % of membrane-bound and internalized activities.

### 2.9 Inoculation of Mice with Dual Xenografts

The *in vivo* animal studies were performed under a protocol approved by the UCSF Institutional Animal Care & Use Committee (IACUC). Using precisely similar protocol from our prior report, homozygous (nu/nu) athymic male mice of 5–6 weeks old (Jackson Laboratories or Envigo-Harlan Laboratories, Livermore CA) inoculated with PC3-Pip (left flank, 3 million cells) and PC3-Flu (right flank, 2.5 million cells) dual xenografts.^10^ Around 100–200 mm^3^ tumor size was perceived after 1–2 weeks postinoculation.

### 2.10 *In Vivo* PET Imaging and Biodistribution Studies

Nine days postinoculation, when the tumor size reached 100–200 mm^3^, the animals were anesthetized using 2% isoflurane, and the respective ^89^Zr-radiolabeled starPEG nanocarriers were administered *via* tail vein (∼7.4 MBq in 100 μL of saline per mouse). The study population included three groups (*n* = 4 mice per group). The mice were scanned at 24, 48, 168, and 216 h post radiopharmaceutical injection in a μPET/CT imaging system (Inveon, Siemens Medical Solutions, Malvern, PA). PET data were acquired for 20 min at 24 h and 48 h, 30 min at 168 h, and 40 min at 216 h in list mode, and the manufacturer’s two-dimensional (2D) ordered subsets’ expectation maximization (OSEM) algorithm was used to reconstruct the data. The imaging data were then normalized to the injected activity to parameterize images to %ID/cc. The imaging data was processed in open-source AMIDE software (http://amide.sourceforge.net/). The tumor-bearing mice were sacrificed at 216 h postinjection of the ^89^Zr-radiolabeled starPEG nanocarriers. Blood was collected through a cardiac puncture, and major organs (liver, kidney, spleen, heart, pancreas, lung, brain, femur, muscle, testis, and subcutaneous tumor) were harvested. Blood and major organs were weighed and analyzed in an automated γ counter (Hidex, Turku, Finland). The percent injected dose per gram of tissue (%ID/g) was determined by comparing standard radioactivity.

### 2.11 Autoradiography

After analyzing the dissected organ samples in the γ counter (Hidex), the tumors were embedded in optimal cutting temperature (OCT) compound and flash frozen on dry ice. Using a microtome, the frozen tumor tissues were sectioned at a thickness of 20 μm and mounted on iQID charged-particle digital autoradiography imaging systems (QScint Imaging Solutions, LLC, Tucson, AZ). The raw autoradiography data were processed in ImageJ software.

### 2.12 Statistical Analysis

All data are presented as mean ± standard deviation in plots. The data were subjected to Student’s *t*-test (unpaired, two-tailed, equal variance) for statistical analysis. Differences at the 95% confidence level (*P* < 0.05) are considered to be statistically significant.

## 3 Results

### 3.1 Design, Synthesis, and Radiolabeling of starPEG Conjugates

#### 3.1.1 Design of starPEG Conjugates

**Figure 2:**
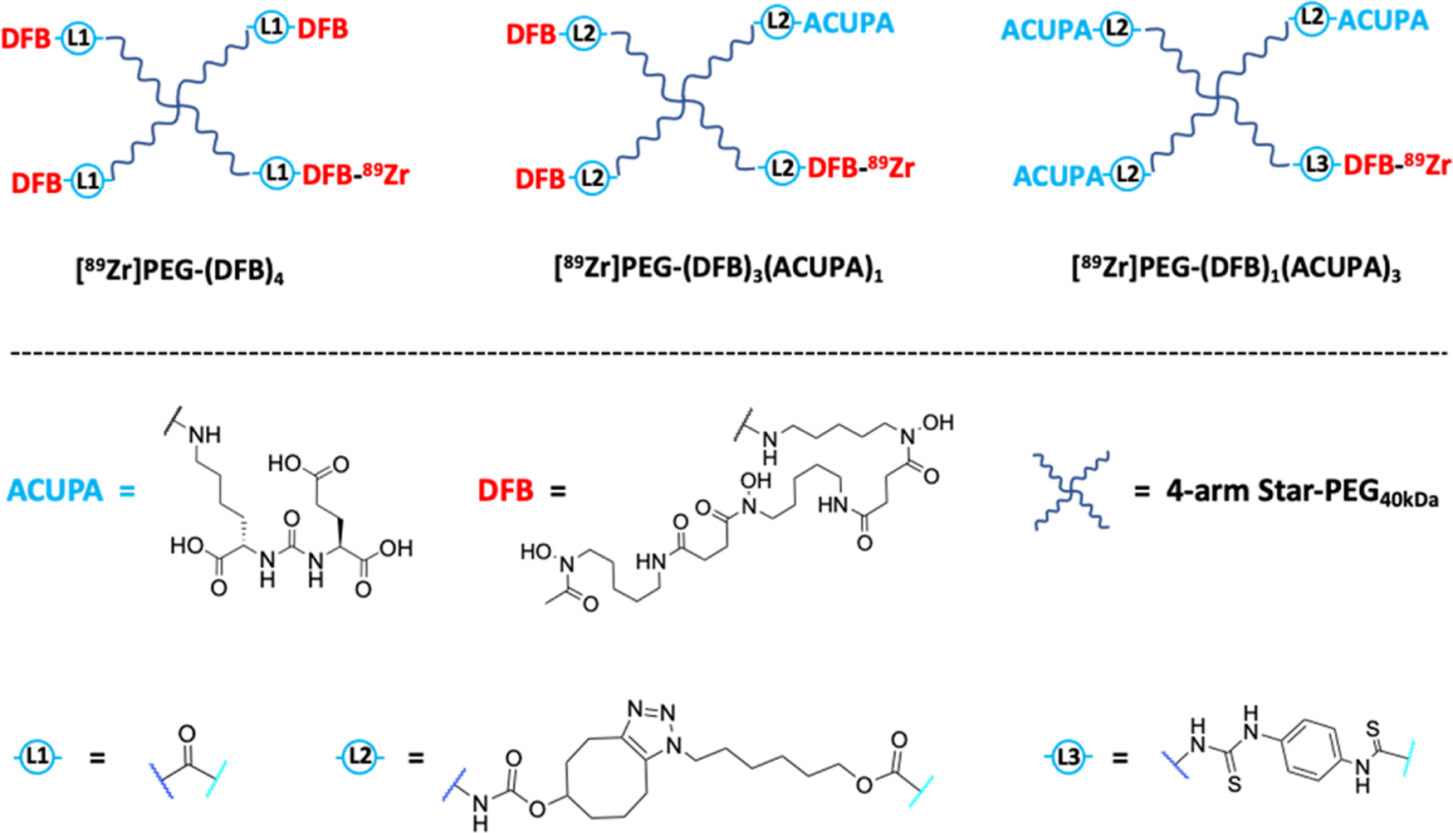
Representative chemical structures of ^89^Zn-labeled starPEG nanocarriers to evaluate the PSMA-targeted PET imaging of prostate cancer. [^89^Zr]PEG-(DFB)_4_, previously reported nontargeted nanocarrier, was used in this study as a baseline control.^25^

We hypothesized that adding PSMA-targeting ACUPA ligands would increase tumor accumulation of the nanocarriers. To test this hypothesis, two starPEGs conjugated with one or three PSMA-targeting ACUPA ligands were designed and synthesized, and their PSMA-targeting ability with tissue penetration was compared with a nontargeted congener without any ACUPA ligands (Figure 2).^25^ All of the starPEG conjugates were tethered with deferoxamine B (DFB) ligands, which is a robust ^89^Zr chelator commonly used in the development of PET imaging agents.^35^ The PEG polymer of 40 kDa, 15 nm, a Food and Drug Administration (FDA) approved polymer for safe human use,^39−41^ was used to synthesize the nanocarriers.

#### 3.1.2 Synthesis and Characterization of starPEG Conjugates

**Scheme 1:**
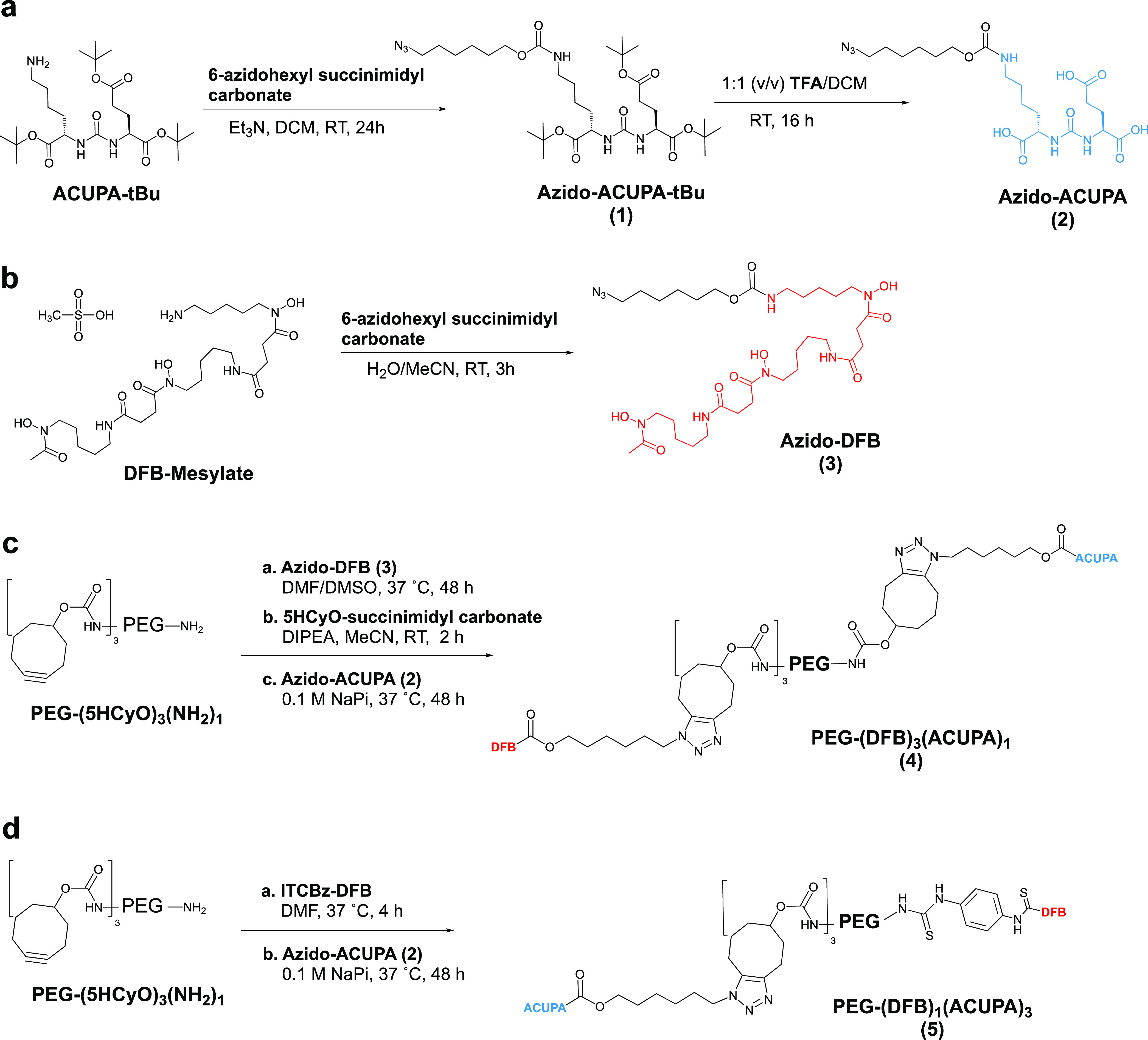
Synthesis of the Azido Derivatives (a) Azido-ACUPA, (b) Azido-DFB, and Their Conjugation to starPEG Nanocarriers to Produce (c) PEG-(DFB)_3_(ACUPA)_1_ and (d) PEG-(DFB)_1_(ACUPA)_3_ Detailed synthetic routes with chemical structures have been provided in the Supporting Information. Individual synthetic steps with intermediate structures for the targeted nanocarriers have been presented in Supporting Information Schemes S1 and S2.

PEG-DFB_4_ was synthesized by the reaction of 4-armed PEG succinimidyl carbonate with DFB mesylate following our previously reported synthetic route.^25,34^ The two starPEG conjugates, PEG-(DFB)_3_(ACUPA)_1_ (**4**) and PEG-(DFB)_1_(ACUPA)_3_ (**5**), were synthesized using second-generation azide click reactions with cyclooctyne (Schemes 1c,d and S1 and S2).^42^ The azide counterparts Azido-ACUPA (**2**) and Azido-DFB (**3**) were synthesized following reported procedures (Scheme 1a,b).^25,43,44^ The previously synthesized and reported PEG-(5HCyO)_3_(NH_2_)_1_ was utilized as the starting material for the targeted nanocarriers.^25^ PEG-(5HCyO)_3_(NH_2_)_1_ conjugate with free amine was treated with Azido-DFB (**3**) to form PEG-(DFB)_3_(NH_2_)_1_ (**S1**) and was subsequently treated with 5-hydroxycyclooctyne (5HCyO)-succinimidyl carbonate, followed by Azido-ACUPA (**2**), to produce PEG-(DFB)_3_(ACUPA)_1_ (**4**), whereas PEG-(5HCyO)_3_(NH_2_)_1_ was reacted with isothiocyanatobenzyl-DFB (ITCBz-DFB) to produce PEG-(5HCyO)_3_(DFB)_1_ (**S3**), which was further reacted with Azido-ACUPA (**2**) to yield PEG-(DFB)_1_(ACUPA)_3_ (**5)**. Detailed synthetic schemes with the respective chemical structures of the polymer conjugates are provided in the supplementary information (Schemes S1 and S2). ^1^H and/or ^13^C NMR were recorded for the newly synthesized ligands and starPEG conjugates (Figures S1–S11). The click conjugation of Azido-ACUPA (**2**) and Azido-DFB (**3**) to the cyclooctyne counterpart of starPEGs was confirmed by the peaks at 4.33 and 4.01 ppm corresponding to the CH_2_ protons close to triazole and carbamate groups in the linkers, respectively (Figure S8). However, none of these peaks at 4.33 and 4.01 ppm were observed in PEG-(DFB)_4_ as it does not bear any linker with triazole and carbamate groups. Though the peak position of PEG-(DFB)_3_(ACUPA)_1_ (**4**) and PEG-(DFB)_1_(ACUPA)_3_ (**5**) looks identical, different numbers of DFB ligands could be seen by the relative peak integrals at 3.17, 2.80, and 2.49 ppm corresponding to DFB ligands to that of other peaks in the aliphatic region (Figures S5–S7). Moreover, the conjugation of DFB through a *p*-isothiocyanatobenzyl linker was confirmed by the aromatic proton signal at 7.35 ppm. Additionally, HRMS of the small molecule intermediates, including Azido-ACUPA-tBu, Azido-ACUPA, and Azido-DFB ligands, was also recorded (Figures S12–S14).

#### 3.1.3 Radiolabeling of starPEG Conjugates

The radiolabeling was carried out by treating the respective starPEG conjugates with ^89^Zr-oxalate. The resulting complex was purified in a PD-10 size-exclusion desalting column by eluting with saline solution.^34^ The yield of the ^89^Zr radiolabeling was 90–95% by iTLC analysis (Figure S15). The isolated yields were 93–98% for PEG-(DFB)_4_ (*n* = 3) and PEG-(DFB)_3_(ACUPA)_1_ (*n* = 2) and 81–82% (*n* = 2) for radiolabeled PEG-(DFB)_1_(ACUPA)_3_ based on starting ^89^Zr (Table S1). The specific activities ranged from 31.1–44.8 MBq/mg for PEG-(DFB)_4_, 31.8–46.3 MBq/mg for PEG-(DFB)_3_(ACUPA)_1_, and 26.3–39.8 MBq/mg for PEG-(DFB)_1_(ACUPA)_3_.

### 3.2 *In Vitro* Cell-Binding Assay

#### 3.2.1 Competition Radioligand Binding Assay

**Figure 3:**
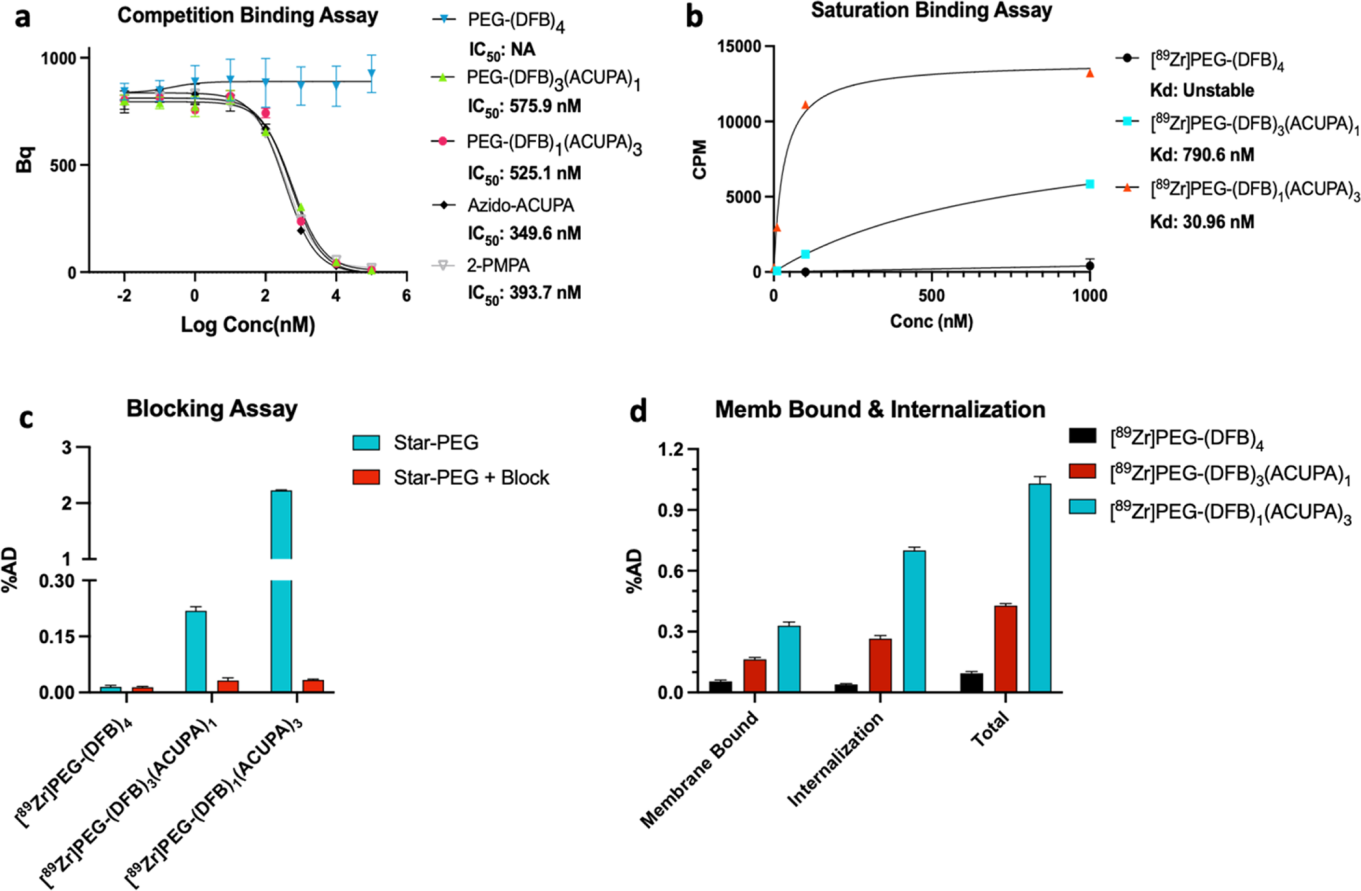
Cell-binding assays with starPEG nanocarriers in PSMA+ PC3-Pip and PSMA– PC3-Flu cell lines demonstrate efficient cell binding and uptake of PSMA-targeted nanocarriers. (a) IC_50_ of nonradiolabeled starPEGs, Azido-ACUPA, and 2-PMPA determined by ^68^Ga-PSMA-11-based *in vitro* competitive radioligand binding assay in PSMA+ PC3-Pip cells (^NS^*P* > 0.05, Student’s *t*-test). (b) *K*_d_ measurement of ^89^Zr-labeled starPEGs in the PSMA+ PC3-Pip cell line by a saturation binding assay. (c) Blocking assay of ^89^Zr-labeled starPEGs (100 nM) in PSMA+ PC3-Pip cells using PSMA-2 as the blocking agent at 1 h (%AD = percentage added dose). Detailed blocking assays at different concentrations and incubation times are presented in the Supporting Information (Figures S17–S19). (d) Membrane-bound and internalization assay of the ^89^Zr-labeled starPEGs at 1 h in PSMA+ PC3-Pip cells (%AD = percentage added dose). The membrane-bound activity was collected by 5 min of acid wash with a cold mixture of 50 mM glycine and 150 mM NaCl. Membrane-bound and internalization assays at later time points are presented in the Supporting Information (Figure S20).

The relative binding affinity of the nonradiolabeled starPEG nanocarriers was obtained in a competitive radioligand binding assay using ^68^Ga-PSMA-11 (Figure 3a and Table S2).^44,45^ 2-PMPA and the intermediate Azido-ACUPA were used as positive controls.^46^ As expected, the nontargeted nanocarrier PEG-(DFB)_4_ did not show any indication of specific binding in PSMA+ PC3-Pip cells. However, the IC_50_ values for the targeted nanocarriers were found to be 517 ± 58 nM for PEG-(DFB)_3_(ACUPA)_1_ and 526 ± 1.3 nM for PEG-(DFB)_1_(ACUPA)_3_ with 95% confidence interval in the range of 359–706 and 404–683 nM, respectively. This demonstrated an insignificant difference (^NS^*P* > 0.05) in the competitive binding affinity of these targeted nanocarriers despite the presence of different numbers of PSMA-targeting ACUPA ligands. A similar IC_50_ was obtained for Azido-ACUPA (349.6 nM) and 2-PMPA (393.7 nM) with 95% confidence interval in the range of 290–420 and 182–482 nM, respectively.

#### 3.2.2 Saturation Binding Assay

The binding affinity of the ^89^Zr-labeled nanocarriers was further evaluated in a saturation binding assay in PSMA+ PC3-Pip cells that demonstrated ∼25-fold lower dissociation constant for [^89^Zr]PEG-(DFB)_1_(ACUPA)_3_ (*K*_d_ = 30.96 nM) with three copies of ACUPA ligands compared to [^89^Zr]PEG-(DFB)_3_(ACUPA)_1_ (*K*_d_ = 790.6 nM) with only one copy of ACUPA ligands (Figure 3b and Table S3). In contrast, no indication of specific binding was witnessed in the nontargeted [^89^Zr]PEG-(DFB)_4_ nanocarrier.

#### 3.2.3 Blocking Assay

Further, a binding and blocking assay was performed using PSMA-2 as the blocking agent at 1, 4, and 24 h time points and different nanocarrier concentrations (Figures 3c and S16–S19).^47−49^ The targeted nanocarriers, [^89^Zr]PEG-(DFB)_3_(ACUPA)_1_ and [^89^Zr]PEG-(DFB)_1_(ACUPA)_3_, demonstrated higher uptake selectively in PSMA+ PC3-Pip cells, and the uptake was significantly reduced in the presence of the known PSMA binder PSMA-2.^47^ In comparison, no specific uptake of the nanocarriers was perceived in PSMA– PC3-Flu cells. Noticeably, at lower probe concentrations (10 and 100 nM), [^89^Zr]PEG-(DFB)_1_(ACUPA)_3_ with three copies of ACUPA ligands demonstrated exceptionally high PSMA-targeted cell uptake compared to [^89^Zr]PEG-(DFB)_3_(ACUPA)_1_ with just one copy of ACUPA (Figure S17). However, as the concentration of the nanocarriers was increased to 1000 nM, the ratio of uptake to block in PSMA+ PC3-Pip cells decreased, which demonstrated relatively higher nonspecific cell uptake at higher concentrations, presumably due to saturation of the binding sites (Figure S18). Similarly, an increase in the nonspecific cell uptake was also observed at higher time points (Figure S19).

#### 3.2.4 Membrane-Bound and Internalization Assay

Next, we tested the degree of cellular uptake and internalization. The membrane-bound and internalized activities were isolated in PSMA– PC3-Flu and PSMA+ PC3-Pip cells by acid wash (an ice-cold mixture of 150 mM sodium chloride and 50 mM glycine) at different time points (Figures 3d and S20). Significantly higher membrane-bound activities were observed for the targeted nanocarriers in PSMA+ PC3-Pip cells, which remained almost similar over time up to 24 h (Figure S20a). The internalized activities for the targeted nanocarriers increased steadily from 1 to 24 h (Figure S20b). Overall, the nanocarrier with three copies of PSMA-targeting ACUPA ligands demonstrated higher membrane-bound and internalization than its counterpart with one ACUPA ligand. On the contrary, no evidence of PSMA-targeted cell uptake was observed for the nontargeted nanocarrier [^89^Zr]PEG-(DFB)_4_. Taken together, these results demonstrate efficient cell binding and internalization for the PSMA-targeted nanocarriers, with relatively higher affinity and cellular uptake when comparing [^89^Zr]PEG-(DFB)_1_(ACUPA)_3_ to [^89^Zr]PEG-(DFB)_3_(ACUPA)_1_.

### 3.3 *In Vivo* μPET/CT Imaging

**Figure 4:**
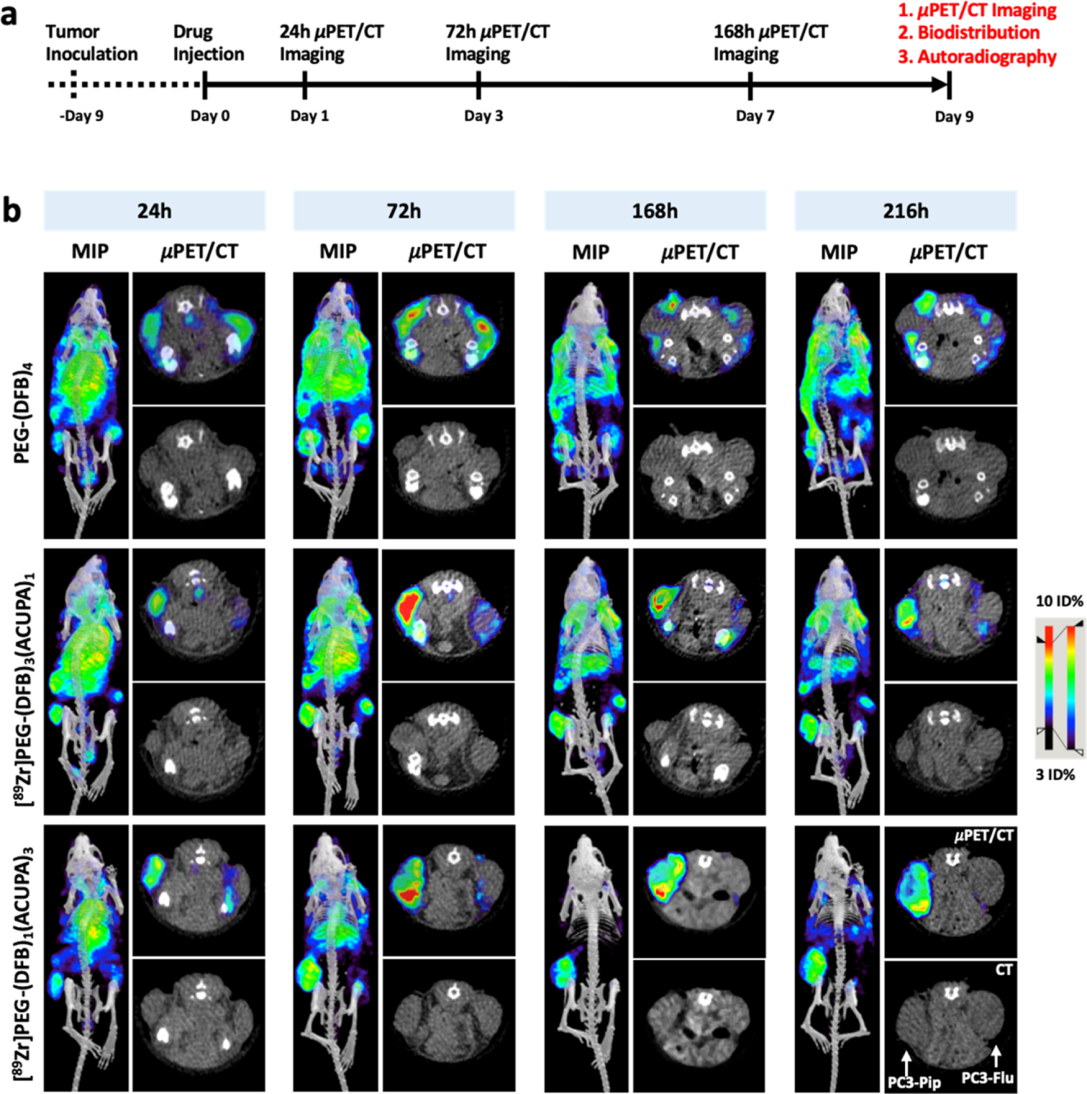
*In vivo* μPET/CT imaging. (a) Representation of experimental design for *in vivo* evaluation of the ^89^Zr-labeled starPEGs in mice bearing dual xenografts of PSMA+ PC3-Pip (left flank) and PSMA– PC3-Flu (right flank). (b) Maximum intensity projection (MIP) μPET/CT, axial μPET/CT, and axial CT images obtained at 216 h following administration of ^89^Zr-labeled starPEGs reveal high tumor accumulation with low background tissue retention of [^89^Zr]PEG-(DFB)_1_(ACUPA)_3_ over time. Respective coronal CT and coronal μPET/CT images are presented in the Supporting Information (Figure S21). ROIs on the heart and tumors are presented in the Supporting Information (Figure S22).

*In vivo* μPET/CT imaging of the ^89^Zr-labeled nanocarriers was performed in the nu/nu athymic mice model with subcutaneous dual xenografts of PSMA– PC3-Flu (right flank) and PSMA+ PC3-Pip (left flank). When the tumor size reached 100–200 mm^3^, the mice were administered the ^89^Zr-labeled nanocarriers *via* tail vein and were subjected to multiple time point μPET/CT imaging up to 216 h, as presented in Figure 4a. The study population was comprised of three groups, with one group for each nanocarrier (*n* = 4 mice). The maximum intensity projection (MIP), axial μPET/CT, and CT images of the nanocarriers are presented in Figure 4b, and the respective coronal images are presented in Figure S21. All μPET/CT images were segmented into respective regions of interest (ROI) over heart and tumors (Tables S4–S6), and the time–activity curves were charted (Figure S22).

Overall, starting from 24 h, the targeted nanocarriers, [^89^Zr]PEG-(DFB)_3_(ACUPA)_1_ and [^89^Zr]PEG-(DFB)_1_(ACUPA)_3_, demonstrated significantly increased PSMA-targeted uptake in PSMA+ PC3-Pip to PSMA– PC3-Flu tumors (Figure 4b). In comparison, the nontargeted nanocarrier [^89^Zr]PEG-(DFB)_4_ did not show any difference in the tumor uptake irrespective of PSMA expression with very high background contrast even after 216 h and instead showed higher nonspecific uptake in the skin and subcutaneous soft tissues. The ROI plot demonstrated an increase in tumor accumulation up to 72 h and a mild decrease afterward up to 216 h, which could also be visualized in the μPET/CT images (Figures S21 and S22). Increased background clearance and prominent tumor retention were observed for the targeted nanocarriers in PSMA+ PC3-Pip. However, at 216 h time point, [^89^Zr]PEG-(DFB)_1_(ACUPA)_3_ showed a central tumoral reduction in signal, which could be due to the development of necrosis inside this larger size tumor (Figure 4b).

### 3.4 *Ex Vivo* Organ Biodistribution

**Figure 5:**
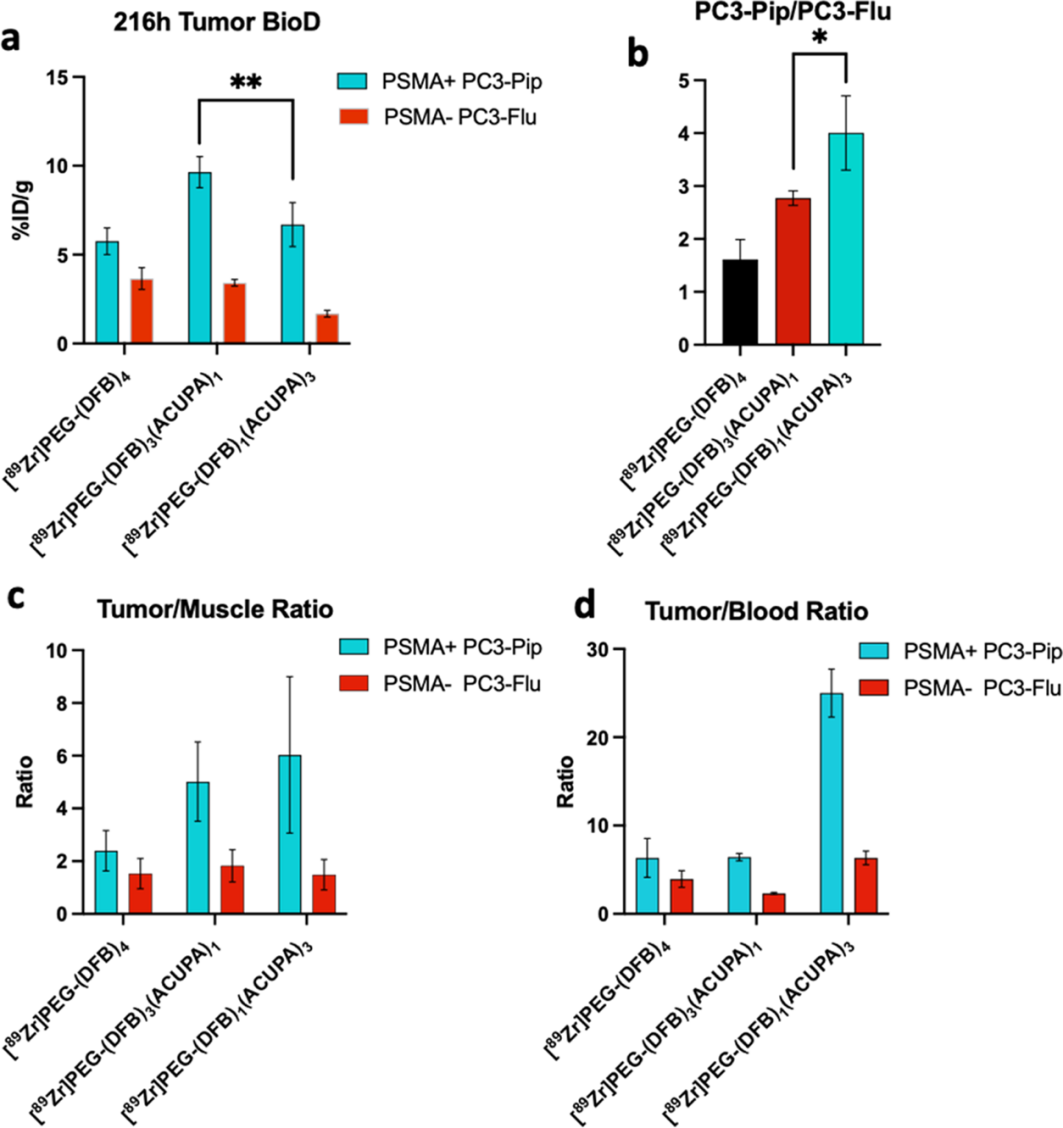
*Ex vivo* organ biodistribution of ^89^Zr-labeled starPEGs. (a) Tumor biodistribution of [^89^Zr]starPEGs at 216 h postinjection of the nanocarriers (*n* = 4, mean ± SD, **P < 0.01, Student’s *t*-test). (b) Ratio of PC3-Pip to PC3-Flu tumor biodistribution of [^89^Zr]starPEGs at 216 h postinjection of the nanocarriers (*n* = 4, mean ± SD, *P < 0.05, Student’s *t*-test). (c) Tumor to the muscle and (d) tumor to blood of [^89^Zr]starPEGs at 216 h postinjection of the nanocarriers. *Ex vivo* biodistribution of ^89^Zr-labeled starPEGs on selected major organs is presented in the supporting information (Figure S23).

The dual xenografts bearing mice were sacrificed after the 216 h time point μPET/CT imaging, and the major organs including tumors were collected to quantify the distribution of ^89^Zr-labeled nanocarriers. The *ex vivo* organ biodistribution results are presented in Figures 5 and S23 and Tables S7 and S8. Interestingly, 9.64 ± 0.87%ID PSMA+ PC3-Pip tumor uptake was observed for [^89^Zr]PEG-(DFB)_3_(ACUPA)_1_, which was significantly higher (**P < 0.01) than the uptake (6.69 ± 1.24%ID) obtained for [^89^Zr]PEG-(DFB)_1_(ACUPA)_3_. However, despite greater PC3-Pip tumor uptake of [^89^Zr]PEG-(DFB)_3_(ACUPA)_1_, a significantly higher PC3-Pip/PC3-Flu ratio (*P < 0.05) was noted for [^89^Zr]PEG-(DFB)_1_(ACUPA)_3_, demonstrating high PSMA-targeted uptake of the later. On the other hand, the nontargeted [^89^Zr]PEG-(DFB)_4_ demonstrated 5.75 ± 0.74%ID uptake in PC3-Pip tumors but failed to provide a higher PC3-Pip/PC3-Flu ratio, consistent with EPR-based nonspecific uptake. While the PC3-Pip/muscle ratios of both the targeted nanocarriers were comparable at around 5–6, the PC3-Pip/blood ratio of [^89^Zr]PEG-(DFB)_1_(ACUPA)_3_ was significantly greater, 25 compared to 6, than DFB_4_ and ACUPA_1_ starPEGs (Figure 5c,d). Overall, while the PSMA+ PC3-Pip tumor accumulation of both the targeted nanocarriers is comparable, [^89^Zr]PEG-(DFB)_1_(ACUPA)_3_ possessed highly improved PSMA+ PC3-Pip to background contrast to that of other nanocarriers without or with one PSMA-targeting ACUPA ligands.

### 3.5 Autoradiography Analysis

**Figure 6:**
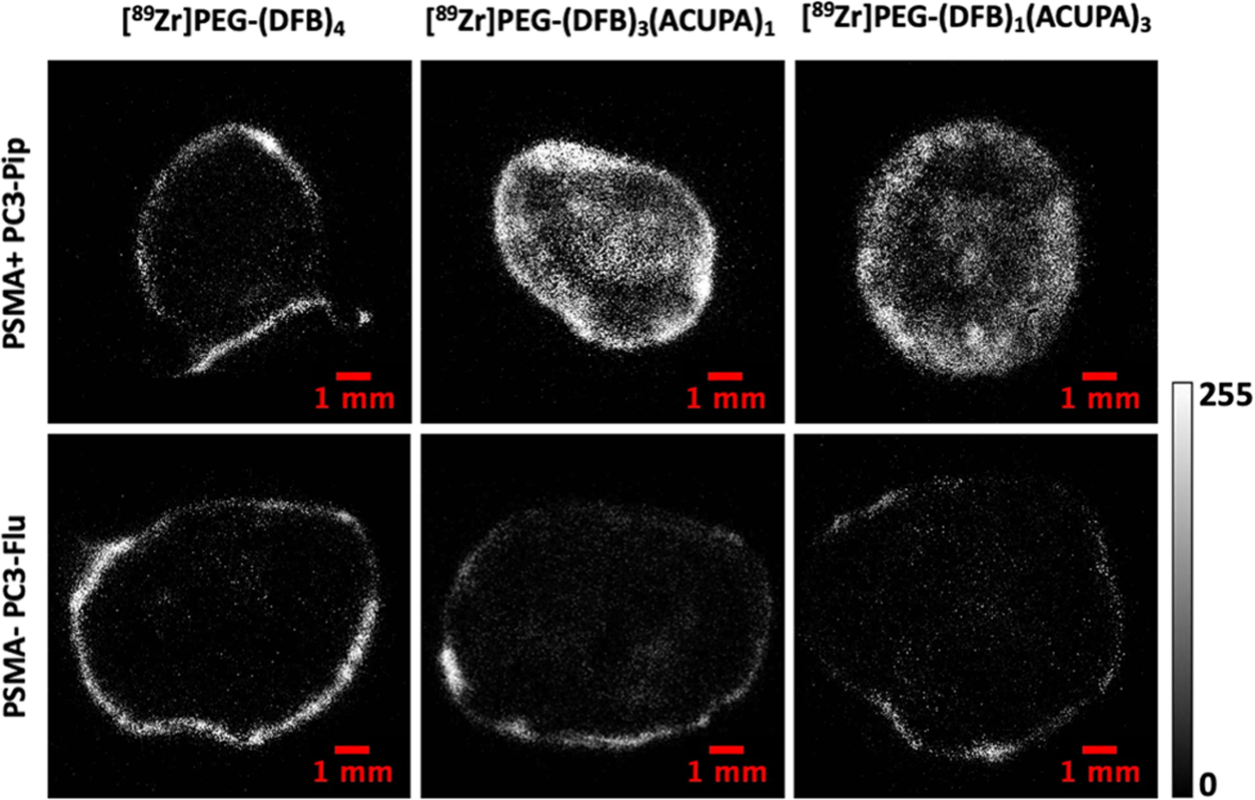
Autoradiography images of 20 μm tumor slices of PSMA+ PC3-Pip and PSMA– PC3-Flu tumors collected after 216 h postinjection of ^89^Zr-labeled nanocarriers.

The tumors dissected at 216 h postinjection were subjected to autoradiography analysis to explore the distribution of the ^89^Zr-labeled nanocarriers inside the bulk tumor tissue (Figure 6). Both targeted nanocarriers with one or three PSMA-targeted ACUPA ligands demonstrated excellent uptake with deep tumor penetration in the PC3-Pip tumors. In contrast, only peripheral accumulation of the nontargeted nanocarrier [^89^Zr]PEG-(DFB)_4_ was seen in the PC3-Pip tumor. Irrespective of the presence of PSMA-targeted ACUPA ligands, all of the nanocarriers demonstrated only peripheral accumulation in the PC3-Flu tumors. Moreover, the distribution of the targeted nanocarriers in PC3-Pip tumors was not homogenous, which could be due to the specific tumor vasculature and/or central necrosis of the large-size tumor.

## 4 Discussion

In this study, we report two newly designed ^89^Zr-labeled starPEG_40kDa_ nanocarriers with one or three copies of ACUPA ligands (Figure 2) and evaluated their PSMA-targeted imaging and deep tumor penetrability in PSMA– PC3-Flu and PSMA+ PC3-Pip prostate cancer xenografts. The nanocarriers were tethered to ^89^Zr chelator DFB ligands for PET imaging.^35^ The pharmacokinetics of both the ^89^Zr-labeled PSMA-targeted nanocarriers were compared with our previously reported nontargeted nanocarrier starPEG_40kDa_ without any ACUPA ligands. In our prior study, ^89^Zr-DFB_4_-starPEG demonstrated very high passive accumulation and retention (>10%ID 9 days postinjection) in MX-1 and HT-29 tumor models, indicative of high EPR.^25^

The 4-armed starPEG nanocarrier of 40 kDa molecular weight (15 nm hydrodynamic diameter) provides an optimal size for EPR-based tumor accumulation with an extended half-life.^25,26^ However, it has been well established that, along with the nanocarrier size, the pharmacokinetics of EPR-mediated passive uptake is strongly influenced by tumor vasculature permeability and macrophages.^27,30,31^ A nontargeted nanostar polymer demonstrated high and homogeneous tumor accumulation (14.8%ID/g) in CT26 tumors with highly leaky vasculature.^30^ However, the same nanostar polymer was unable to penetrate deep into the poorly leaky BxPC3 tumor and mostly accumulated in the tumor periphery (>5.5%ID/g).^30^ Most of the prostate cancer tumor models like PC3, DU-145, and CWR22rv1 human prostate xenografts have been evaluated to be EPR low phenotype with poor deep tumor penetration of those nanocarriers relying on the EPR-mediated passive uptake.^13,30,31^ Herein, we hypothesized that by conjugating the PSMA-targeting ACUPA ligands to the 4-armed starPEG nanocarriers, the target-specific tumor accumulation with deep tissue penetration of the nanocarriers would be improved in prostate cancer xenograft beyond passive uptake.

The targeted nanocarriers were developed by conjugating PSMA-targeted ACUPA ligands to the 4-armed starPEG through cyclooctyne linkers using an azide–cyclooctyne-based metal catalyst-free click reaction (Scheme 1). However, depending on the synthetic convenience, the ^89^Zr chelator DFB was linked to the polymer arms *via* an amide linker in [^89^Zr]PEG-(DFB)_4_, a cyclooctyne triazole linker in [^89^Zr]PEG-(DFB)_3_(ACUPA)_1_, and a p-phenylene thiourea linker in [^89^Zr]PEG-(DFB)_1_(ACUPA)_3_ (Scheme 1). Prior reports demonstrated no marked difference in the pharmacokinetics of the PSMA-targeted probes with distinct linkers associated with the radiometal chelator.^50^ The starPEG nanocarriers were purified by dialysis (12–14 kDa cutoff), and the conjugation of the ligands was confirmed by ^1^H NMR analysis (Figures S4–S8).

^89^Zr radiolabeling of the nanocarriers was performed using the reported protocol that yielded 26.8–46.3 MBq/mg specific activity with around 81–98% (isolated) radiolabeled yield (Figure S15 and Table S1). The specific activity and isolated yield of PEG-(DFB)_4_ and PEG-(DFB)_3_(ACUPA)_1_ were relatively higher than those of PEG-(DFB)_1_(ACUPA)_3_, which could be rationalized with the number of DFB ligands conjugated to the nanocarrier. Overall, three 4-armed PEG-based nanocarriers without or with different numbers of ACUPA ligands were synthesized and radiolabeled with better specific activity than those of our prior reported starPEG nanocarriers.^25^ The PSMA-targeted *in vitro* cell binding and *in vivo* pharmacokinetics of those polymer nanocarriers were evaluated in PSMA– PC3-Flu and PSMA+ PC3-Pip cells/tumors.

Various cell-binding assays including competition radioligand binding, saturation binding, blocking, and internalization assays were performed to evaluate the PSMA-targeted *in vitro* characteristics of the nanocarriers. Around 25.5-fold lower dissociation constant (*K*_d_) of 30.9 nM was obtained for [^89^Zr]PEG-(DFB)_1_(ACUPA)_3_ with three PSMA-targeted ACUPA ligands to that of [^89^Zr]PEG-(DFB)_3_(ACUPA)_1_ (Figure 3b and Table S3). The blocking, membrane-bound, and internalization assay too demonstrated notably higher PSMA-targeted cell uptake of [^89^Zr]PEG-(DFB)_1_(ACUPA)_3_ with three copies of ACUPA ligands (Figures S17–20). The blocking assay performed at different nanocarrier concentrations demonstrated significantly enhanced binding affinity of [^89^Zr]PEG-(DFB)_1_(ACUPA)_3_ at a lower concentration, compared to when the assay was performed at a higher probe concentration (Figure 3c). These results demonstrated a crucial role of concentration in the binding affinity of the targeted nanocarriers and may be due to a saturation of PSMA binding sites and an increase in the nonspecific cell uptake at higher concentrations. Surprisingly, ^68^Ga-PSMA-11-based competition radioligand binding assay in PSMA+ PC3-Pip cells demonstrated highly comparable IC_50_ (459–575 nM, ^NS^*P* > 0.05) obtained in two independent experiments for both the targeted nanocarriers with one or three ACUPA ligands, respectively (Table S2). It should be noted that the nonradiolabeled nanocarriers with free DFB ligands were evaluated in the competition radioligand binding assay to determine the IC_50_ values. It is thought that the complexation of metal in the chelator could alter the overall charge and hydrophilicity of the nanocarriers and thereby could alter the targeted binding affinity of the nanocarriers.^50−52^ Additionally, few other prior studies on PSMA-targeted probes demonstrated similar inconsistent correlation of the IC_50_ to other *in vitro* binding assay and *in vivo* pharmacokinetics.^13,50^ As expected, no sign of PSMA-targeted binding affinity was witnessed for [^89^Zr]PEG-(DFB)_4_ in any of the *in vitro* cell-binding assays. Overall, it was observed that the conjugation of ACUPA ligands to the polymer nanocarriers strongly influences their PSMA-targeted *in vitro* cell binding affinity and internalization. Moreover, the use of the multivalent PSMA binder [^89^Zr]PEG-(DFB)_1_(ACUPA)_3_ demonstrated increased *in vitro* PSMA binding affinity compared against the single ACUPA containing version. These findings are consistent with other reports utilizing bivalent or multivalent PSMA binders.^13,17,53−57^ Overall, the *in vitro* findings support the use of multivalent PSMA binding to maximize cell binding affinity and uptake.

All of the nanocarriers were subjected to *in vivo m*PET/CT imaging (24, 72, 168, and 216 h) and organ biodistribution post 216 h imaging in the nu/nu athymic mice model implanted with subcutaneous PSMA– PC3-Flu (right flank) and PSMA+ PC3-Pip (left flank) tumors (Figure 4a). Both the targeted nanocarriers [^89^Zr]PEG-(DFB)_3_(ACUPA)_1_ and [^89^Zr]PEG-(DFB)_1_(ACUPA)_3_ demonstrated remarkably high PSMA-targeted uptake in PC3-Pip tumors as compared to the nontargeted [^89^Zr]PEG-(DFB)_4_, which showed only tumor peripheral accumulation irrespective of the tumor type. The imaging and organ biodistribution demonstrated that, as the number of PSMA-targeted ACUPA ligands conjugated to the nanocarriers increased, the ratio of PC3-Pip/PC3-Flu, PC3-Pip/muscle, and PC3-Pip/blood increased significantly (Figure 5). Overall, the background clearance was improved with a targeted accumulation of the nanocarriers in PC3-Pip tumors as the number of ACUPA ligands increased (Figures 4 and 5). It should be noted that, although [^89^Zr]PEG-(DFB)_1_(ACUPA)_3_ demonstrated the highest background clearance and PC3-Pip/blood ratio, [^89^Zr]PEG-(DFB)_3_(ACUPA)_1_ demonstrated relatively higher PC3-Pip uptake (9.64±0.87%ID) (Figure 5 and Table S7). These unpredicted *in vivo* pharmacokinetics of the PSMA-targeted starPEG nanocarriers could be explained by the binding site barrier (BSB) effect, where large-size macromolecules with higher target binding affinity could bind to cells around the periphery of the blood vessels and restrict their further smooth diffusion into the bulk tumors.^13,23,32,33,58^ As demonstrated by Simanek and co-workers, despite increasing the PSMA-targeting motifs from 4 to 64 copies, the targeted tumor uptake of the large-size nanocarriers could get restricted by the BSB effect leading to poor deep tumor penetration.^13^ Other reports also clearly demonstrate that nanocarriers with high target binding affinity might not be very effective for uniform tumor penetration, despite their high *in vitro* cell binding affinity.^33,59−61^ In this present study, it was evident from the *in vitro* cell-binding assay that the increasing number of ACUPA ligands effectively enhanced the PSMA binding affinity of [^89^Zr]PEG-(DFB)_1_(ACUPA)_3_ to that of its counterpart [^89^Zr]PEG-(DFB)_3_(ACUPA)_1_ and could experience a relatively higher BSB effect. Being a relatively weak PSMA binder, [^89^Zr]PEG-(DFB)_3_(ACUPA)_1_ may experience a comparatively low BSB effect to that of [^89^Zr]PEG-(DFB)_1_(ACUPA)_3_ and thus demonstrate higher PSMA-targeted tumor accumulation with tissue penetration. It is interesting to note that the tumor tissue penetration of [^89^Zr]PEG-(DFB)_3_(ACUPA)_1_ appears comparable to that of [^89^Zr]PEG-(DFB)_1_(ACUPA)_3_ (Figure 6). Another possible explanation for the reduction of central tumoral signals could be the development of necrosis in the large-size tumor at later time points. Since the imaging has been performed for up to 9 days, it is difficult to control the tumor size over time, and we observed that the PC3-Pip tumors of the mice group (383 ± 0.051 mg) treated with [^89^Zr]PEG-(DFB)_1_(ACUPA)_3_ were larger than that of the mice group (242 ± 0.054 mg) treated with [^89^Zr]PEG-(DFB)_3_(ACUPA)_1_. Few other reasonable limitations of this study could be a smaller sample size (*n* = 4) and the average size difference between PC3-Pip and PC3-Flu tumors as well.

However, considering the exceptionally higher background clearance and PC3-Pip/blood ratio, [^89^Zr]PEG-(DFB)_1_(ACUPA)_3_ could be more efficient candidates for therapeutic evaluation, for example, to deliver chemotherapeutic or therapeutic radionuclide (^177^Lu, ^225^Ac, etc.) payloads.^9,17,62^ The autoradiography images clearly demonstrated the tissue penetration advantage of the targeted starPEG vs the nontargeted [^89^Zr]PEG-(DFB)_4_ in PC3-Pip and all of the nanocarriers in PC3-Flu where they were unable to penetrate the bulk prostate cancer tumors (Figure 6). Similar to other prostate cancer xenografts like CWR22rv1, DU-145, and PC3, the poorly leaky vasculature and macrophages of PC3-Pip and PC3-Flu could be the primary reason for the EPR-mediated low tumor uptake and tissue penetration.^13,30,31^ Thus, as evaluated in our prior study, the nontargeted [^89^Zr]PEG-(DFB)_4_ demonstrated very high EPR-mediated tumor uptake and tissue penetration in highly leaky MX-1 and HT-29 tumor models but failed in the poorly leaky prostate cancer xenograft evaluated in this study.^25^ Considering the EPR-driven low tumor uptake of those large-size nanocarriers, active targeting of prostate cancer is highly essential to facilitate enhanced tumor uptake with tissue penetration for improved therapeutic efficacy.

## 5 Conclusions

In conclusion, three 4-armed starPEG-based nanocarriers without or with different numbers of ACUPA ligands were synthesized and radiolabeled with good yields. The PSMA-targeted *in vitro* cell binding and *in vivo* pharmacokinetics of the nanocarriers were evaluated in PSMA– PC3-Flu and PSMA+ PC3-Pip cells and xenografts, demonstrating the potential influence of the number of PSMA-targeting ACUPA motifs attached to the nanocarriers. Although both the targeted nanocarriers with one or three copies of ACUPA ligands significantly improved the tumor retention and tissue penetration in PSMA+ PC3-Pip xenografts, the multivalent targeted nanocarrier [^89^Zr]PEG-(DFB)_1_(ACUPA)_3_ with three ACUPA ligands showed a remarkably higher PC3-Pip/blood ratio and background clearance. As expected, the nontargeted [^89^Zr]PEG-(DFB)_4_ demonstrated low EPR-mediated uptake and peripheral accumulation in the poorly leaky PC3-Flu and PC3-Pip xenografts. Overall, PSMA-targeted multivalent polymer nanocarriers significantly improved retention and tissue penetration in xenografts with EPR low phenotypes. The developed multivalent nanocarriers with tumor penetrability and high PC3-Pip to background contrast may be a potential candidate for therapeutic evaluation.

## Supporting Information Available

Synthetic procedures; structural characterization data (^1^H and ^13^C NMR spectra, HRMS); synthetic route to (a) PEG-(DFB)_3_(NH_2_)_1_, (b) PEG-(DFB)_3_(5HCyO)_1_, and (c) PEG-(DFB)_3_(ACUPA)_1_; synthetic route to (a) PEG-(5HCyO)_3_(DFB)_1_, and (b) PEG-(DFB)_1_(ACUPA)_3_; radiolabeling; *in vivo* studies (PDF)

## Abbreviations

PCa: prostate cancer
PSMA: prostate-specific membrane antigen
PEG: poly(ethylene glycol)
PET: positron emission tomography
Ci: curie
tBu: tertiary butyl
DIPEA: *N*,*N*-diisopropylethylamine
ACUPA: ((*S*)-2-(3-((*S*)-5-amino-1-carboxypentyl) ureido) pentanedioic acid
TFA: trifluoroacetic acid
DCM: dichloromethane
FBS: fetal bovine serum
PBS: phosphate-buffered saline
NHS: *N*-hydroxysuccinimide
DCC: *N*,*N*′-dicyclohexylcarbodiimide
iTLC: instant thin-layer chromatography
DFB: deferoxamine B
FDA: Food and Drug Administration
CyO: cyclo octyne
EPR: enhanced permeability and retention
EDTA: ethylenediaminetetraacetic acid
IACUC: Institutional Animal Care & Use Committee
ROI: region of interest
EPR: enhanced permeability and retention
BSB: binding site barrier
