# Supplementary material for: Prostate-Specific Membrane Antigen Targeted Deep Tumor Penetration of Polymer Nanocarriers
Source: ACS Appl Mater Interfaces. 2022 Nov 1;14(45):50569–82. doi: 10.1021/acsami.2c15095 (PMC9673064; doi:10.1021/acsami.2c15095)
Supplement: Supplementary file 1 — am2c15095_si_001.pdf [file am2c15095_si_001.pdf]

Supporting information

Prostate-Specific Membrane Antigen Targeted Deep-Tumor Penetration of Polymer Nanocarriers

Niranjan Meher,[χ] Gary W. Ashley,[†] Anil P. Bidkar,[χ] Suchi Dhrona,[χ] Cyril Fong,[χ] Shaun D. Fontaine,[†] Denis R. Beckford Vera,[χ] David M. Wilson,[χ,§] Youngho Seo,[χ,§] Daniel V. Santi,[†] Henry F. VanBrocklin,[χ,§] and Robert R. Flavell,[,χ,§,¶]*

[χ]Department of Radiology and Biomedical Imaging, University of California, San Francisco, CA 94143, United States.

[†]ProLynx Inc., San Francisco, CA 94158, United States.

[§]Helen Diller Family Comprehensive Cancer Center, University of California San Francisco, San Francisco, CA 94143-0981, United States

[¶]Department of Pharmaceutical Chemistry, University of California, San Francisco, CA 94158-2517, United States

[*]E-mail: robert.flavell@ucsf.edu; Phone: +1-415-353-3638

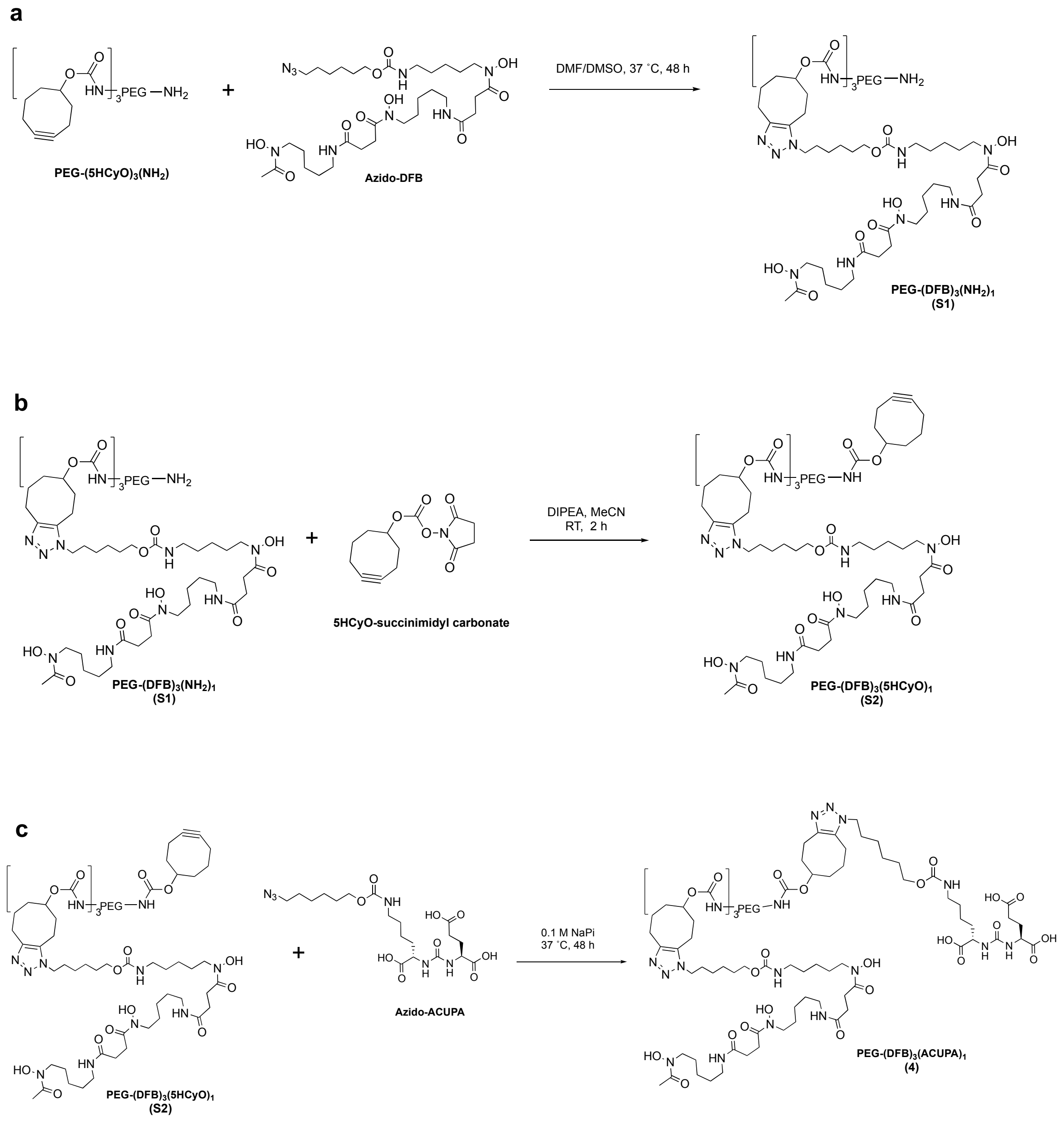

Scheme S1. Synthetic route to (a) PEG-$(DFB)_3(NH_2)_1$, (b) PEG-$(DFB)_3(5HCyO)_1$, and (c) PEG-$(DFB)_3(ACUPA)_1$.

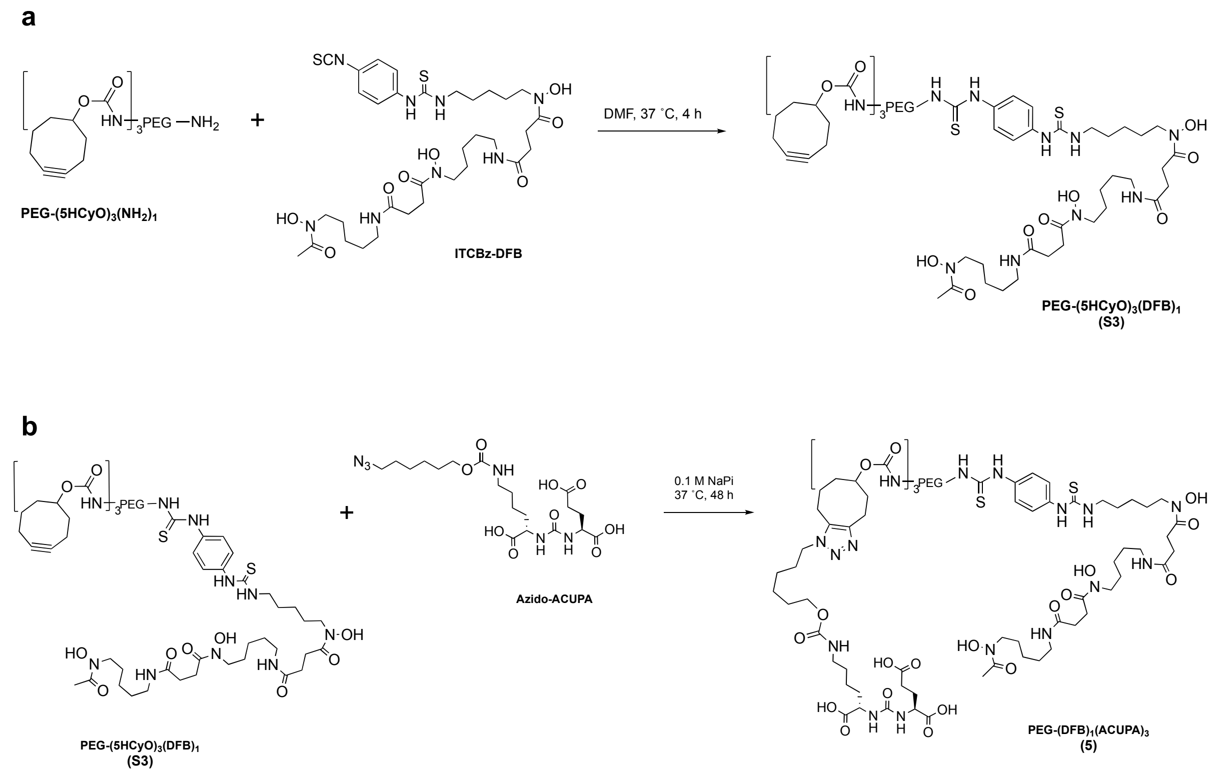

Scheme S2. Synthetic route to (a) PEG-$(5HCyO)_3(DFB)_1$, and (b) PEG-$(DFB)_1(ACUPA)_3$.

3. ^{1}H NMR Spectra

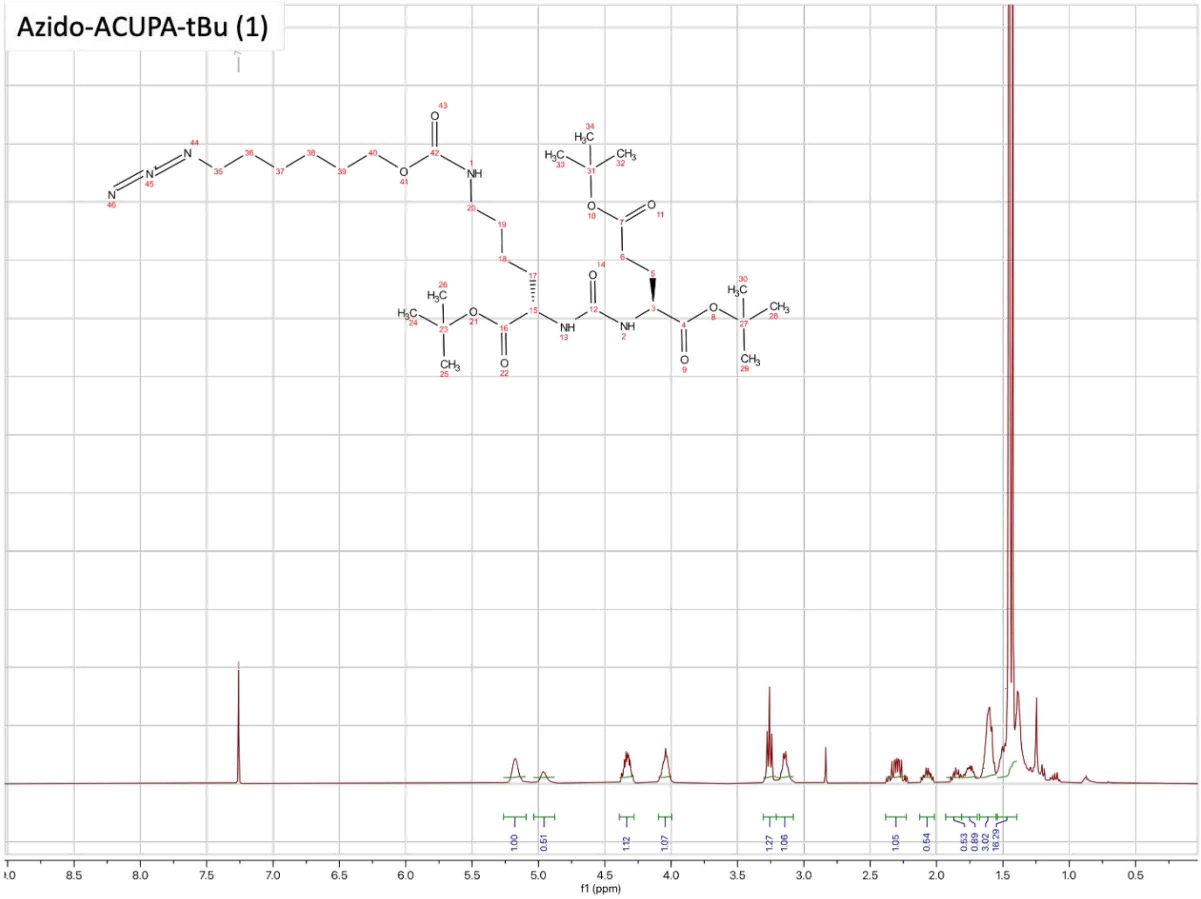

Figure S1. ^{1}H NMR spectra of Azido-ACUPA-tBu.

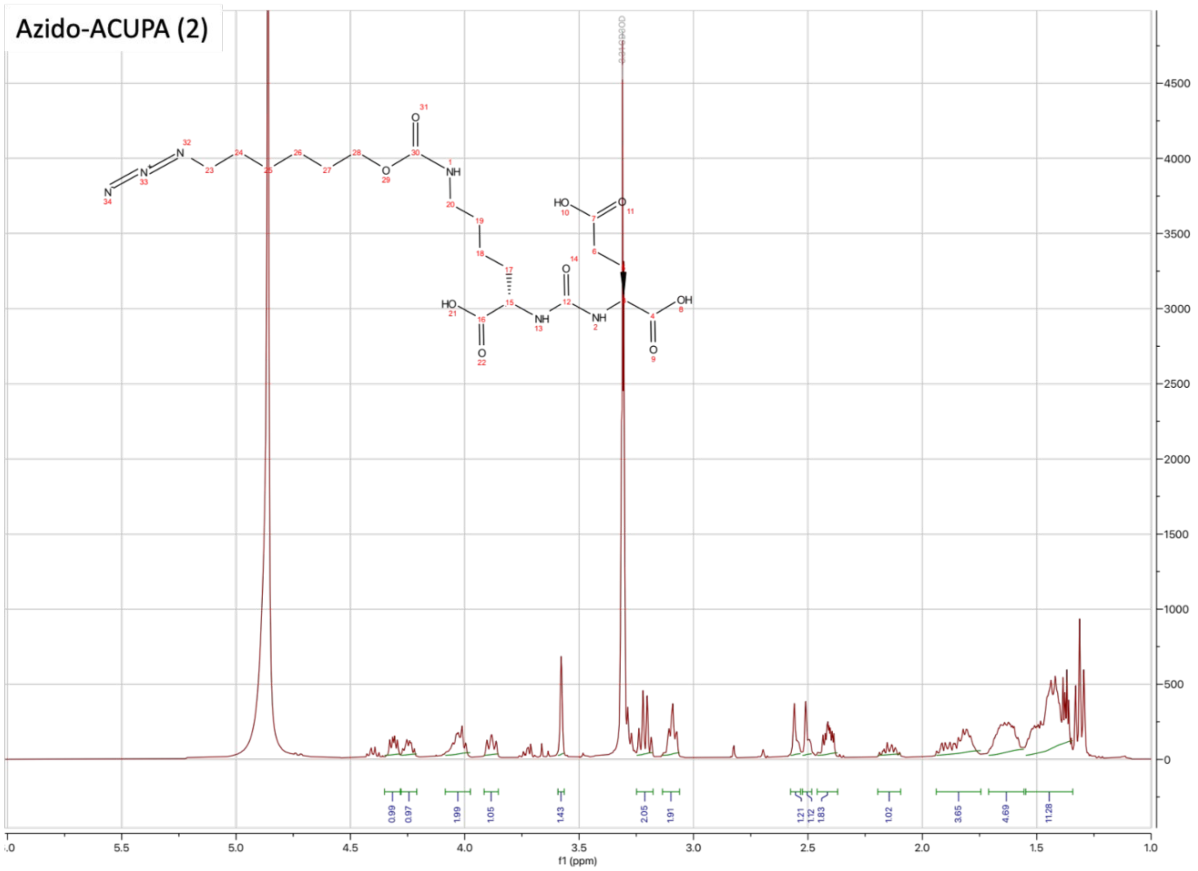

Figure S2. ^{1}H NMR spectra of Azido-ACUPA.

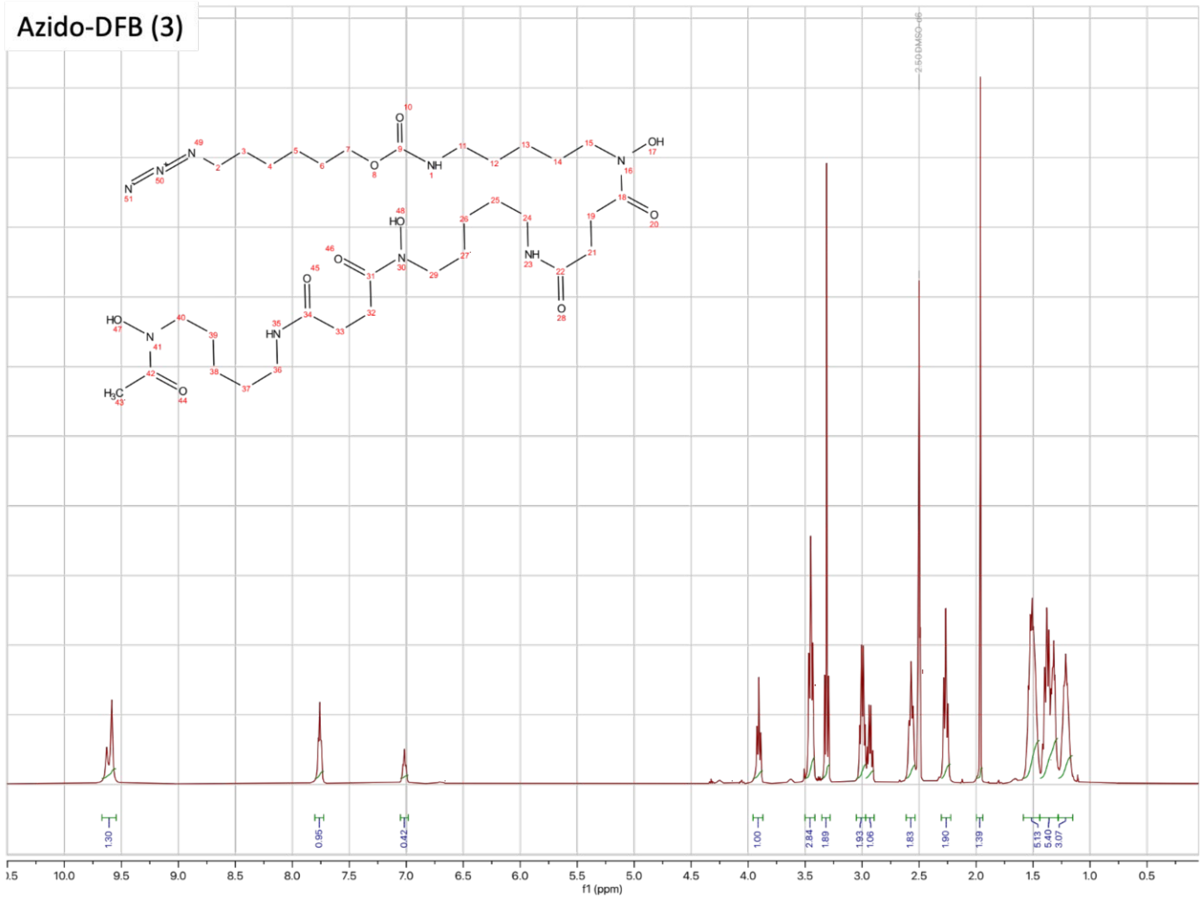

Figure S3. ^{1}H NMR spectra of Azido-DFB.

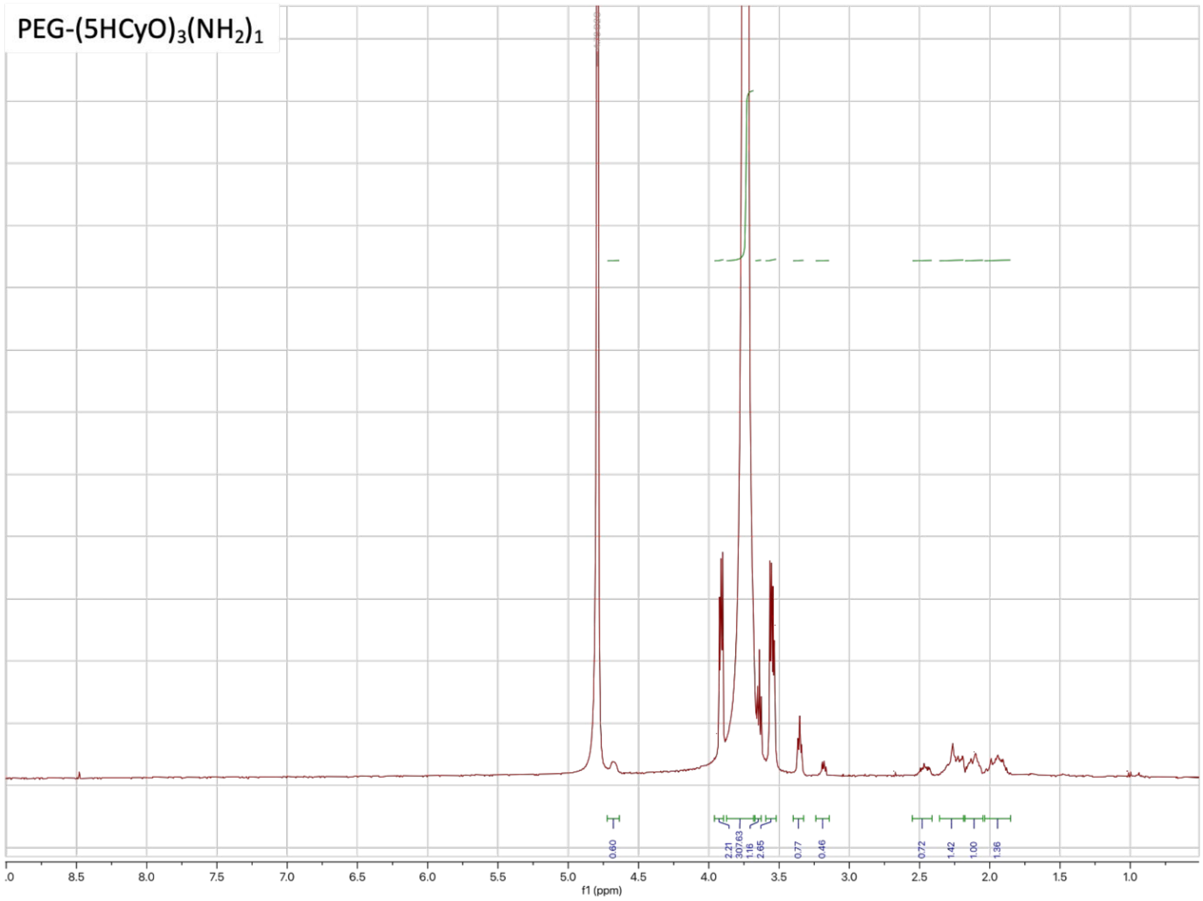

Figure S4. ^{1}H NMR spectra of PEG-$(5HCYO)_3(NH_2)_1$.

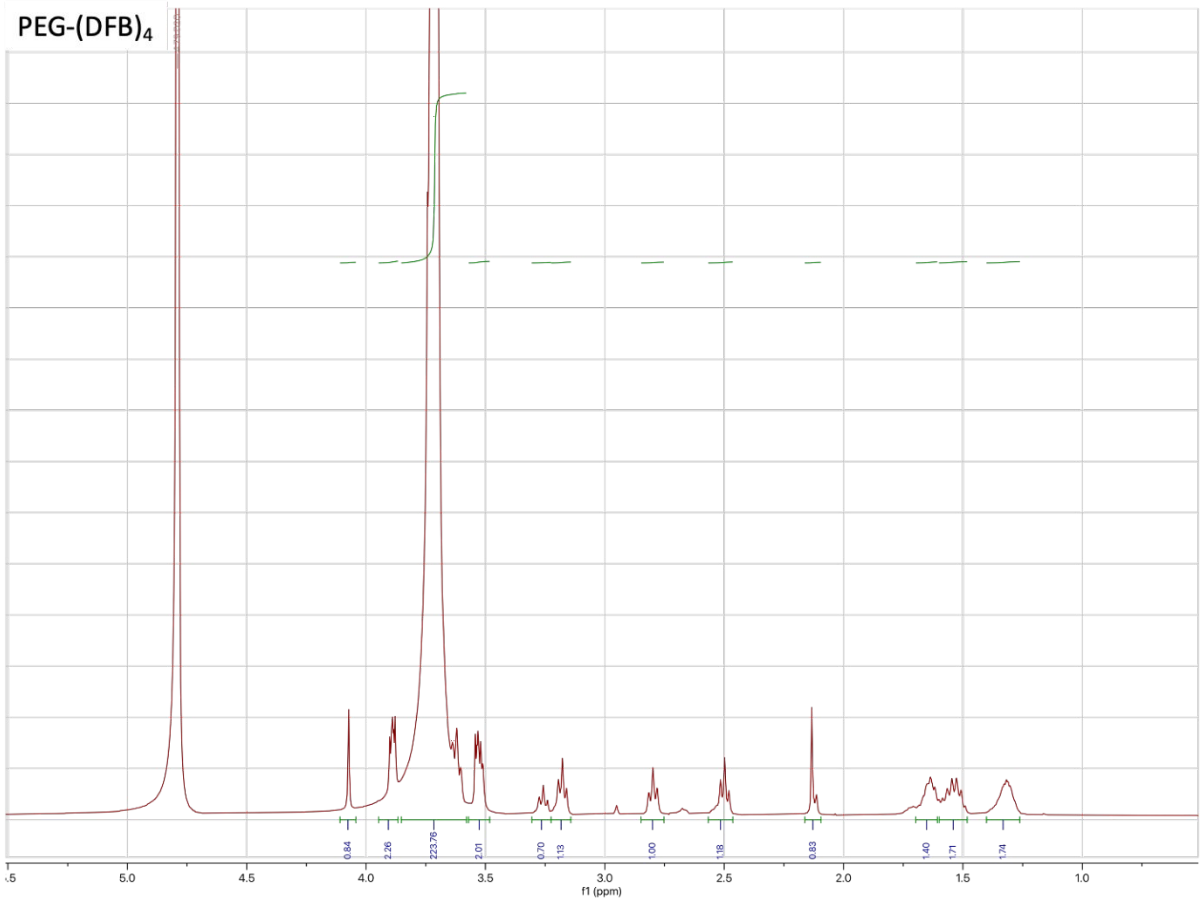

Figure S5. ^{1}H NMR spectra of PEG-$(DFB)_4$.

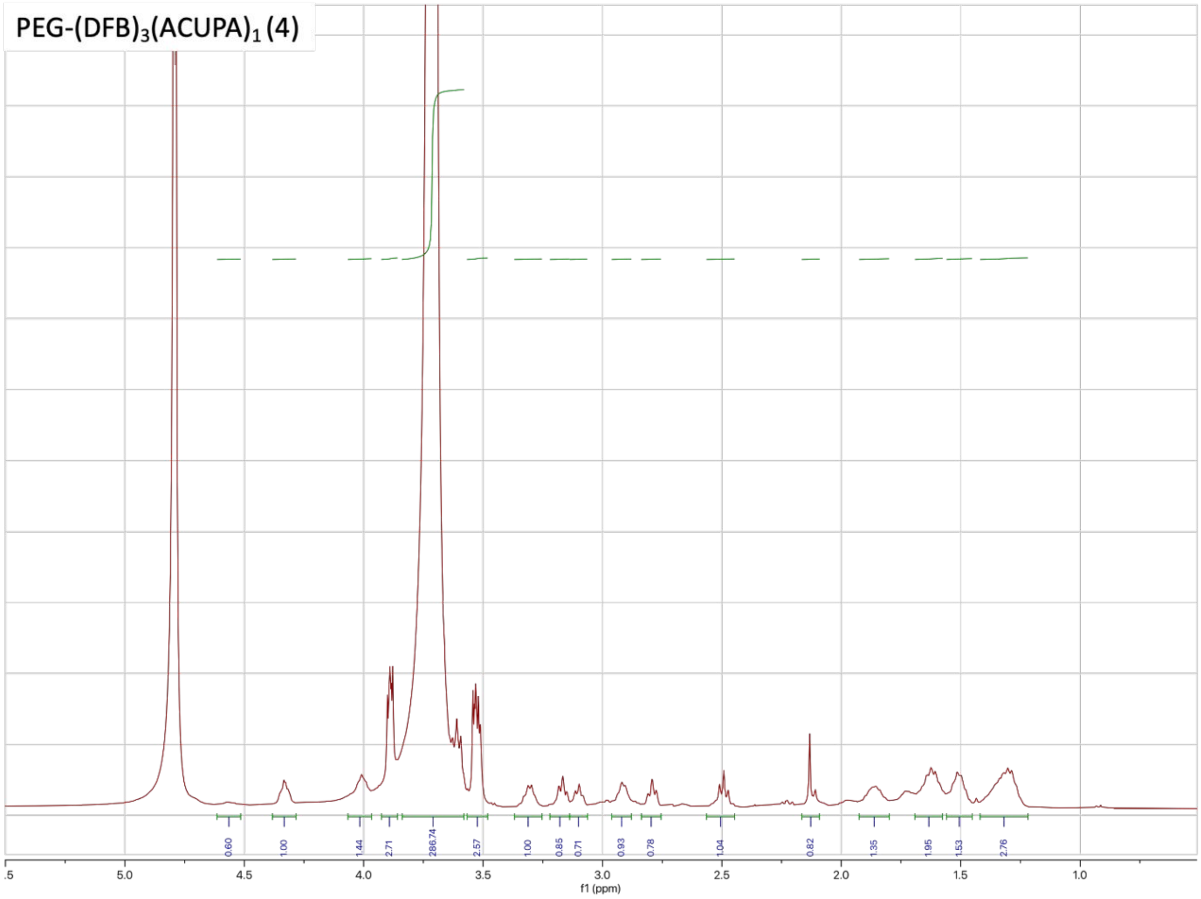

Figure S6. ^{1}H NMR spectra of PEG-$(DFB)_3(ACUPA)_1$.

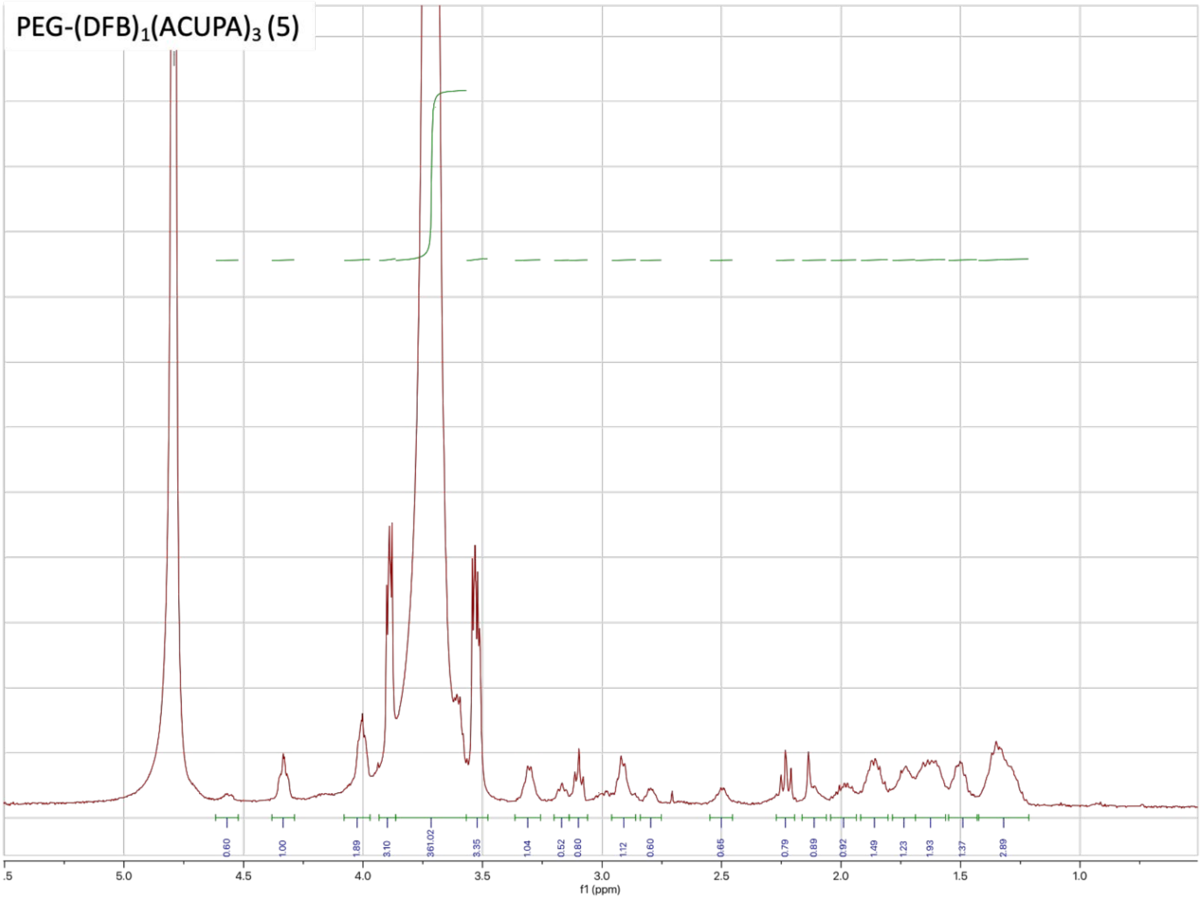

Figure S7. ^{1}H NMR spectra of PEG-$(DFB)_1(ACUPA)_3$.

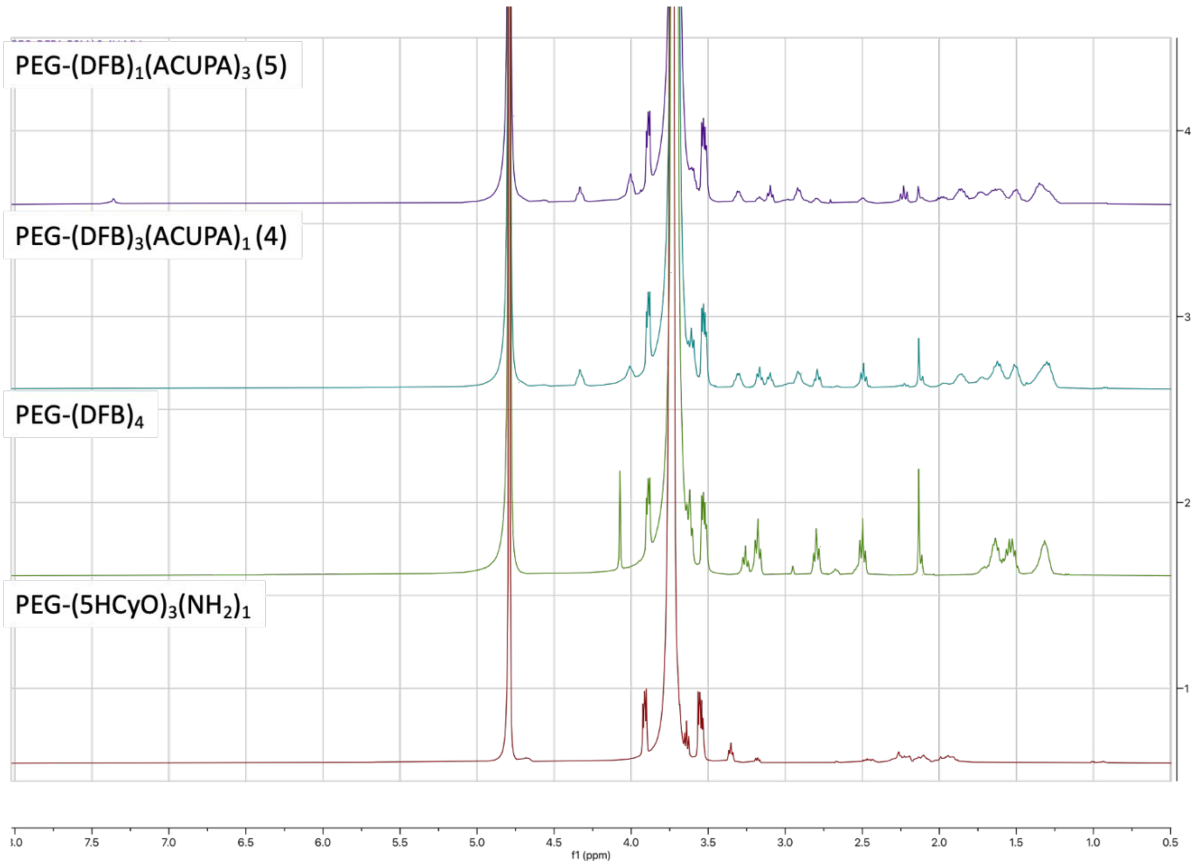

Figure S8. ^{1}H NMR spectra of PEG-$(DFB)_4$, PEG-$(DFB)_3(ACUPA)_1$, and PEG-$(DFB)_1(ACUPA)_3$.

4. ^{13}C NMR Spectra

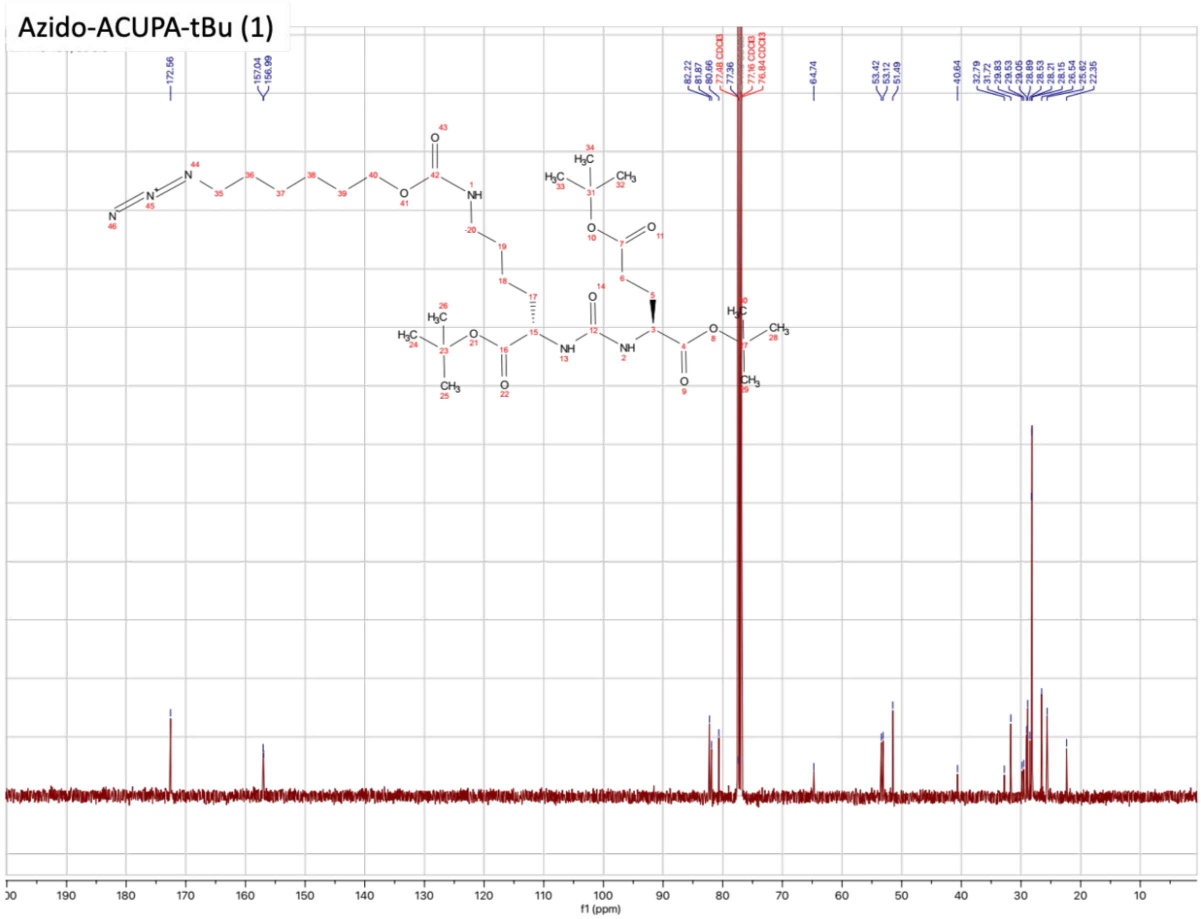

Figure S9. ^{13}C NMR spectra of Azido-ACUPA-tBu.

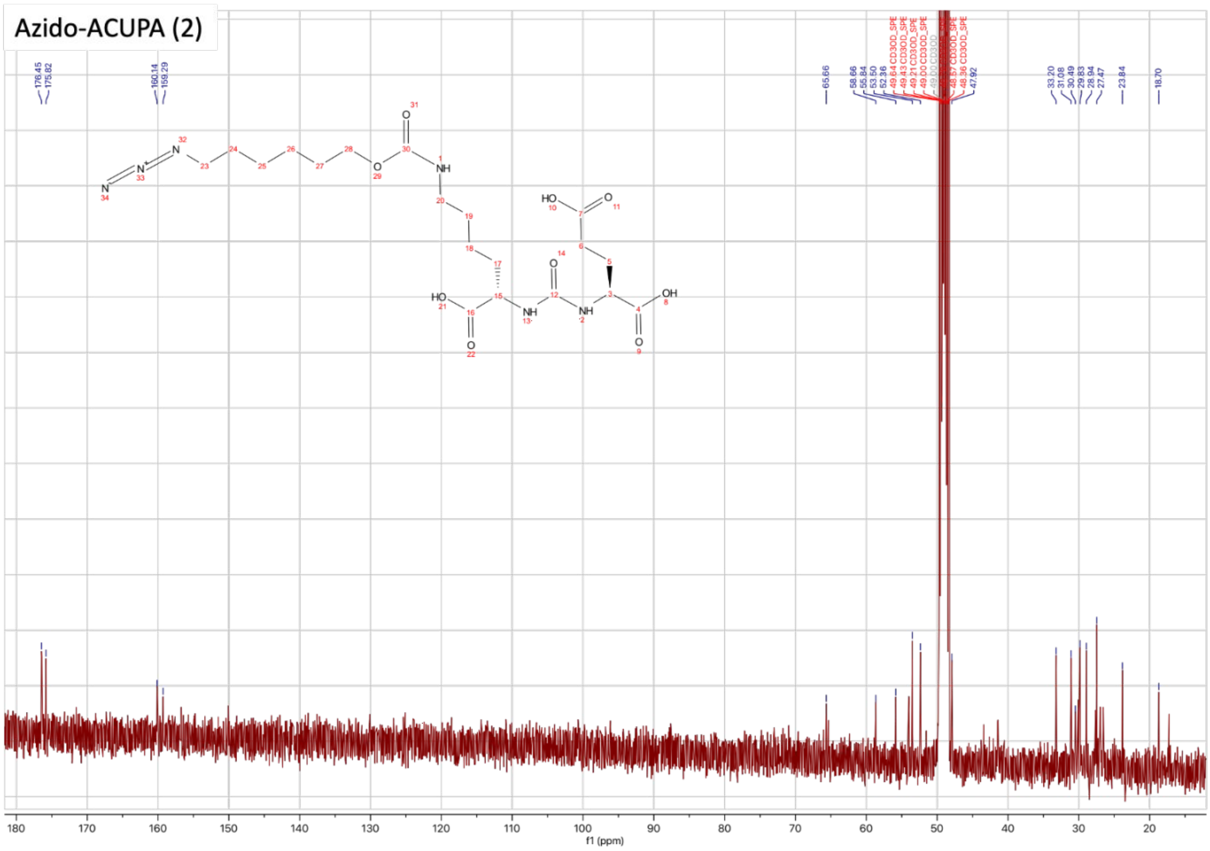

Figure S10. ^{13}C NMR spectra of Azido-ACUPA.

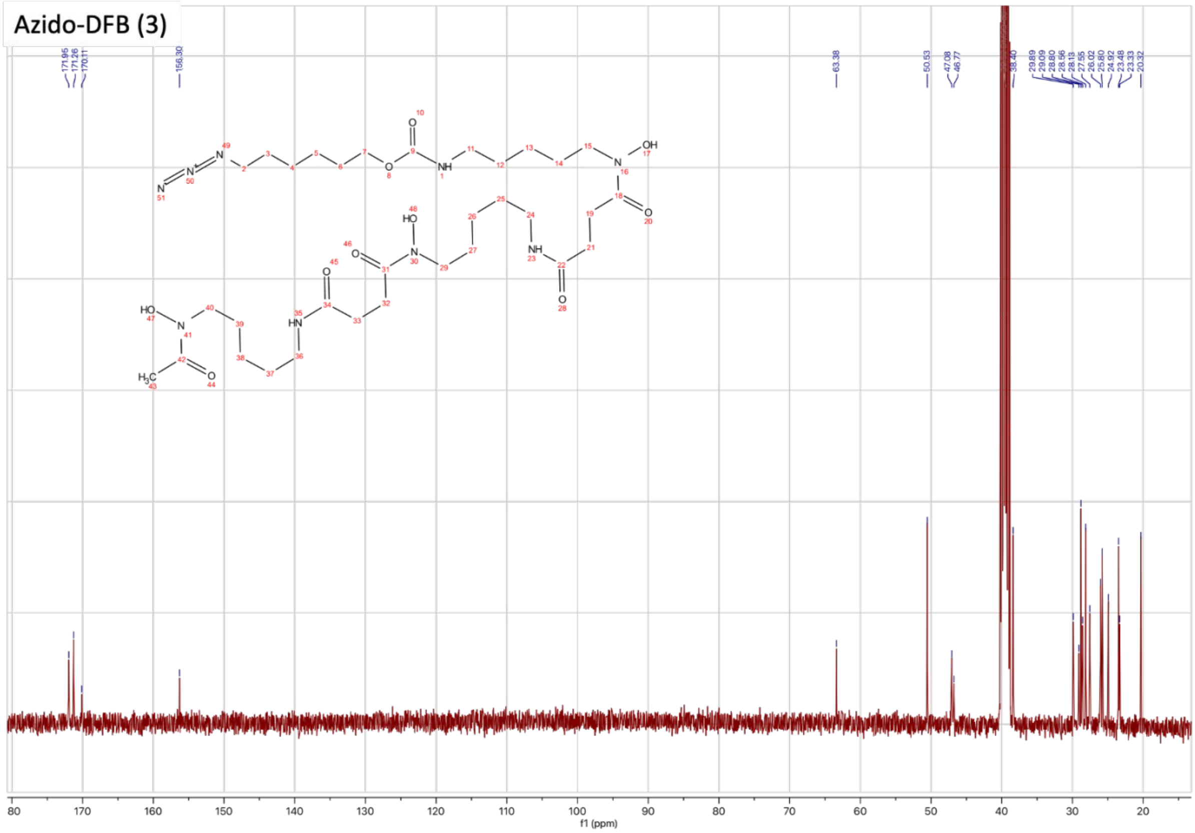

Figure S11. ^{13}C NMR spectra of Azido-DFB.

5. HRMS

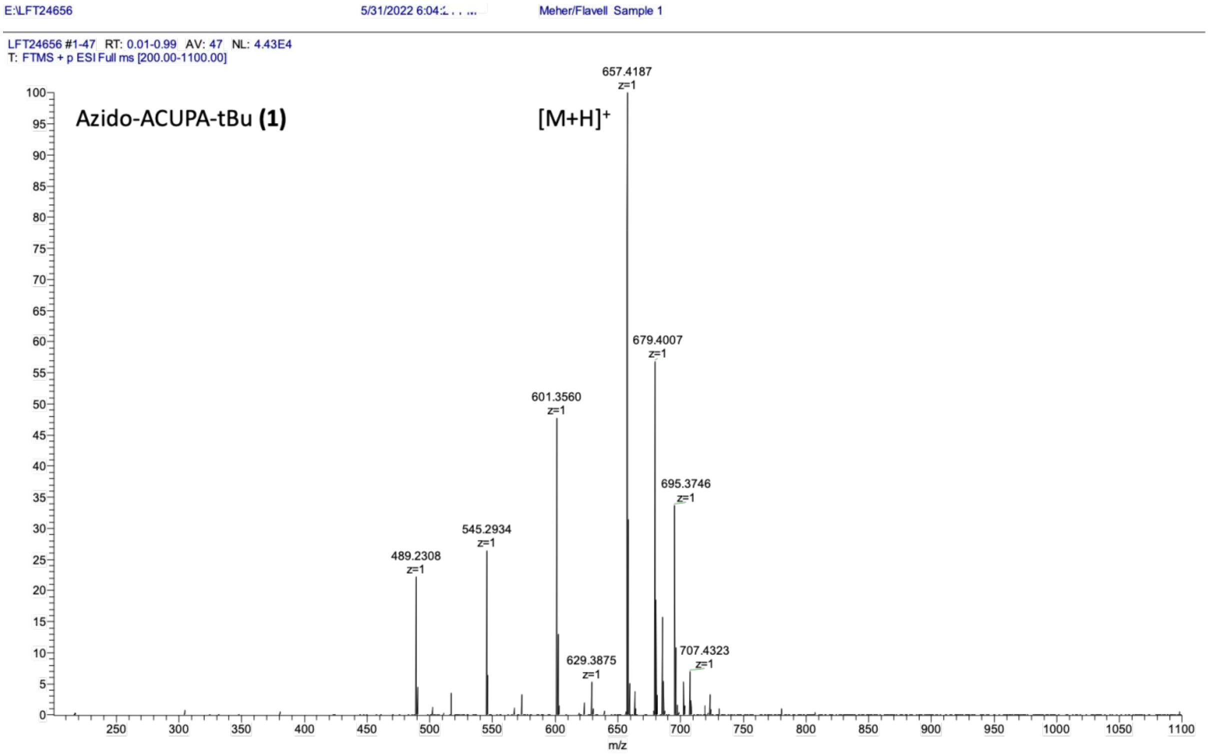

Figure S12. HRMS of Azido-ACUPA-tBu.

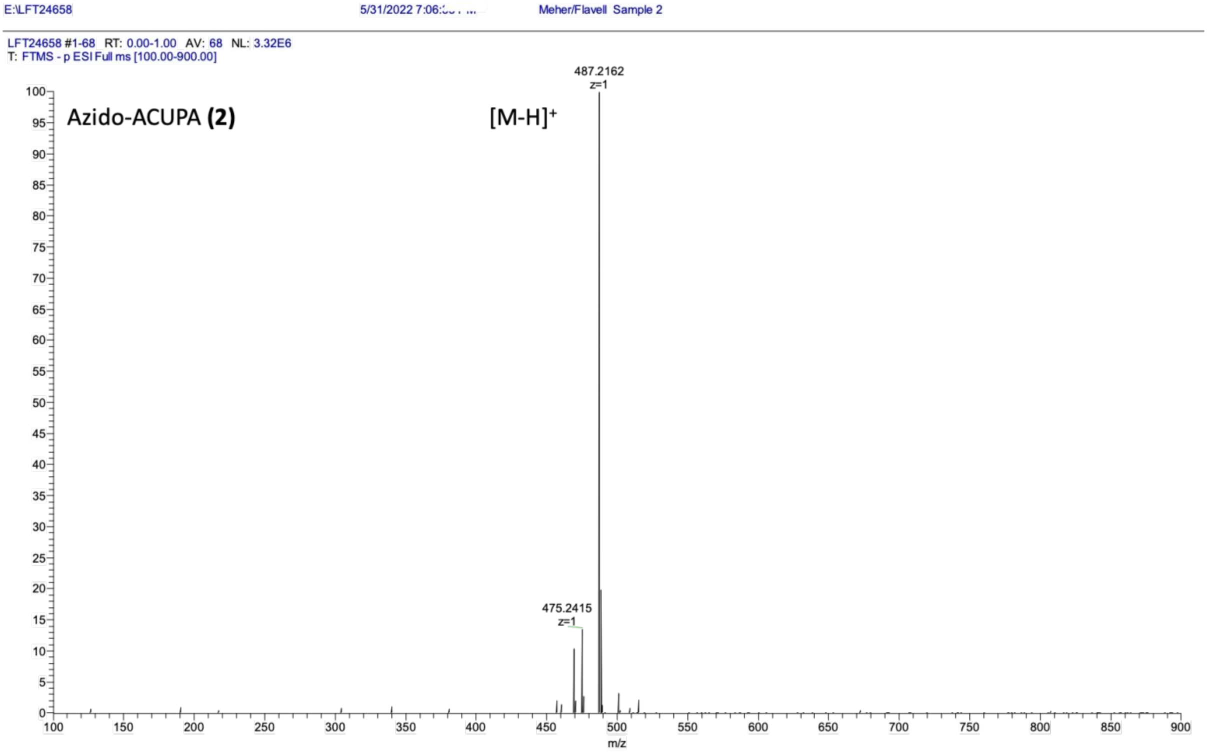

Figure S13. HRMS of Azido-ACUPA.

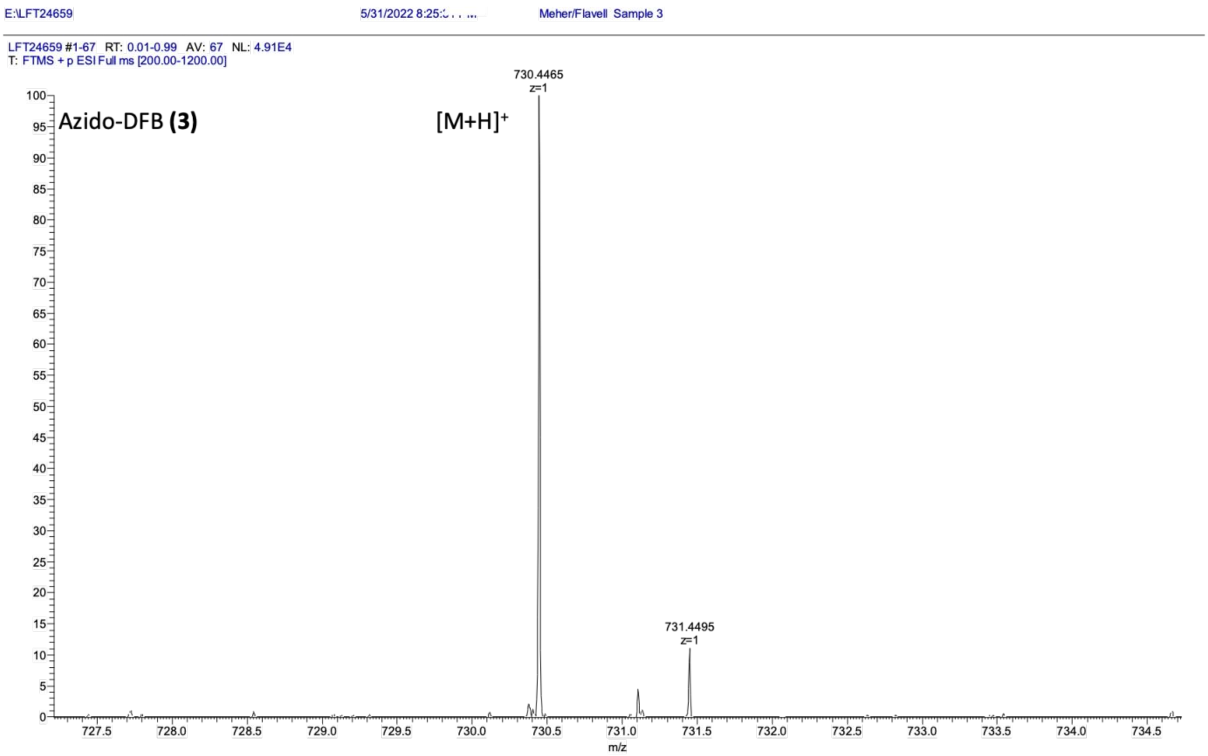

Figure S14. HRMS of Azido-DFB.

6. Radiolabeling

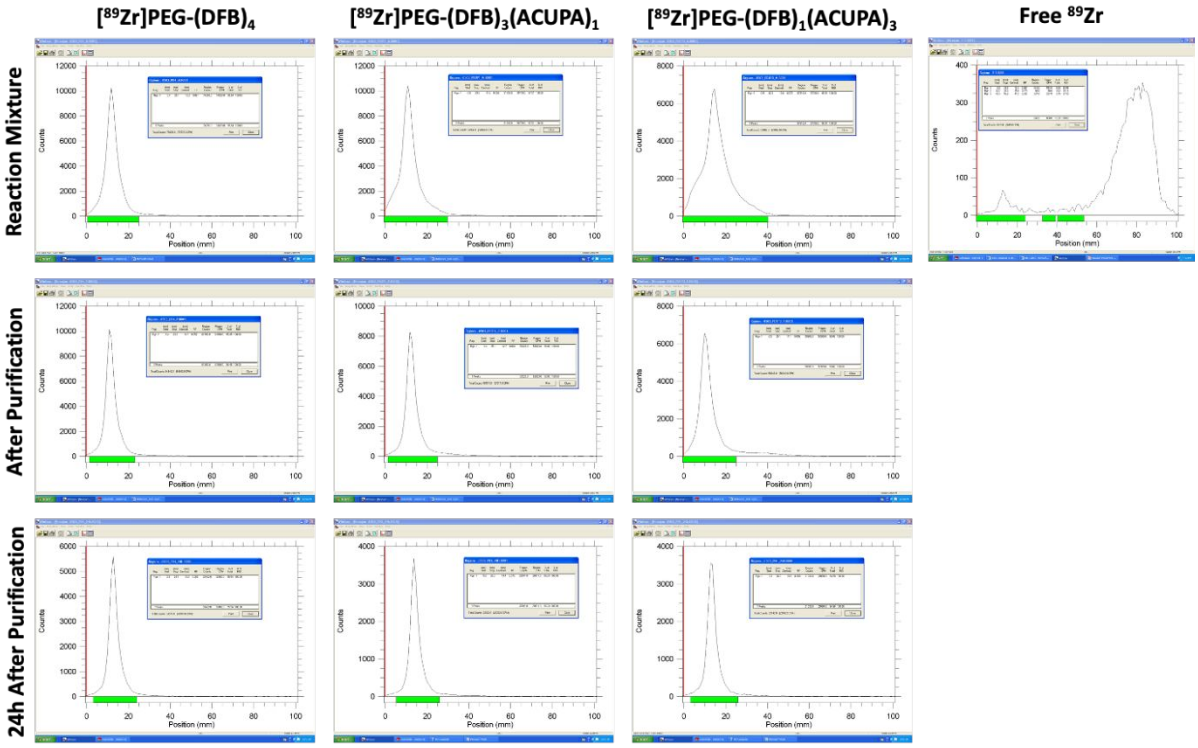

Figure S15. iTLCs of free ^{89}Zr and radiolabeled nanocarrier conjugates before and after purification using glass microfiber chromatography paper impregnated with silica gel and developed using 50 mM EDTA solution as mobile phase. Under these conditions, the radiolabeled nanocarriers stay at the origin, while free radiometal moves to the solvent front.

Table S1. Radiolabeling yield and molar activity ^{89}Zr labeled nanocarriers.

PEG conjugates	Wt (mg)	Activity Taken (MBq)	Isolated Yield (MBq)	Specific Activity (MBq/mg)
PEG-(DFB)4	2.58	91.02	88.43	34.27
	4.0	192.4	179.08	44.77
	4.93	155.77	153.18	31.07
PEG-$(DFB)_3(ACUPA)_1$	2.71	91.02	86.21	31.81
	4.0	192.4	185.0	46.25
PEG-$(DFB)_1(ACUPA)_3$	2.83	91.02	74.37	26.27
	4.0	192.4	159.1	39.77

7. *In vitro* Studies

Table S2. Competition radioligand binding assay results of the unlabeled nanocarriers in PSMA+ PC3-Pip cells using ^{68}Ga-PSMA-11.

Nanocarriers	Experiment 1		Experiment 2	
	IC_{50} (nM)	IC_{50}, 95% CI (nM)	IC_{50} (nM)	IC_{50}, 95% CI (nM)
PEG-$(DBF)_4$	NA	NA	NA	NA
PEG-$(DBF)_3(ACUPA)_1$	575.9	466.3 – 706.6	459.8	359.8 – 584.4
PEG-$(DBF)_1(ACUPA)_3$	525.1	406.4 – 670.7	527.8	404.4 – 683.2
Azido-ACUPA	349.6	290.1 - 420.9	-	-
2-PMPA	393.7	320.3 - 482.4	214.6	182.3 – 253.3

Table S3. Saturation binding assay results of the ^{89}Zr labeled nanocarriers in PSMA+ PC3-Pip cells.

Nanocarriers	K_d (nM)	K_d, 95% CI (nM)	B_{max} (nM)	B_{max}, 95% CI (nM)
[^{89}Zr]PEG-$(DBF)_4$	Unstable	Very Wide	NA	NA
[^{89}Zr]PEG-$(DBF)_3(ACUPA)_1$	790.6	653.2-987.7	10464	9603-11669
[^{89}Zr]PEG-$(DBF)_1(ACUPA)_3$	30.96	26.10-36.69	13934	13427-14455

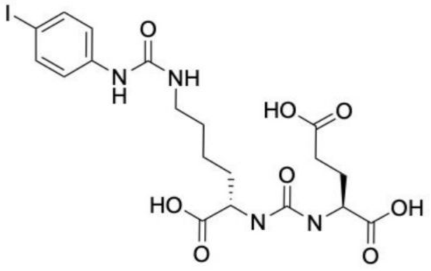

Figure S16. Structure of the PSMA inhibitor ligand used for the in vitro blocking assay (PSMA-2, Ki = 0.24 nM, and IC_{50} = 10 nM).[1-3]

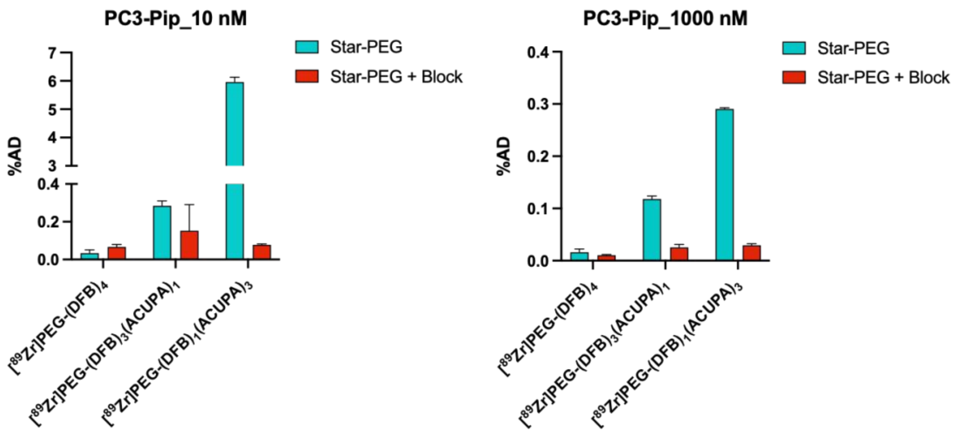

Figure S17. *In vitro* blocking assay of ^{89}Zr labeled nanocarriers at different concentration (10 nm and 1000 nm) at 1 h incubation in PSMA+ PC3-Pip cells by using PSMA-2 as blocking agent.

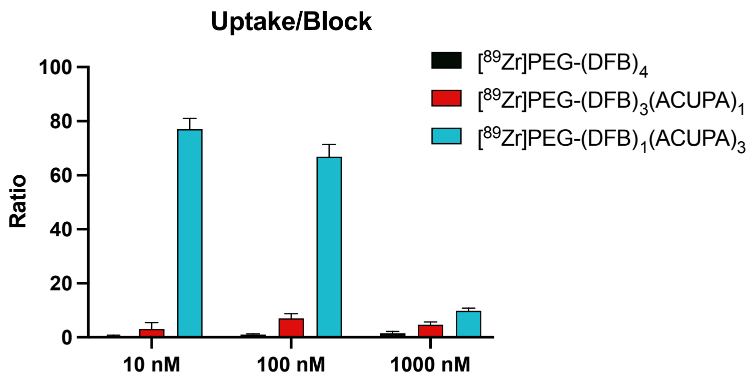

Figure S18. The ratio of uptake/blocking at different concentrations in ^{89}Zr labeled Star-PEGs in PSMA+ PC3-Pip cells by using PSMA-2 as blocking agent at 1 h.

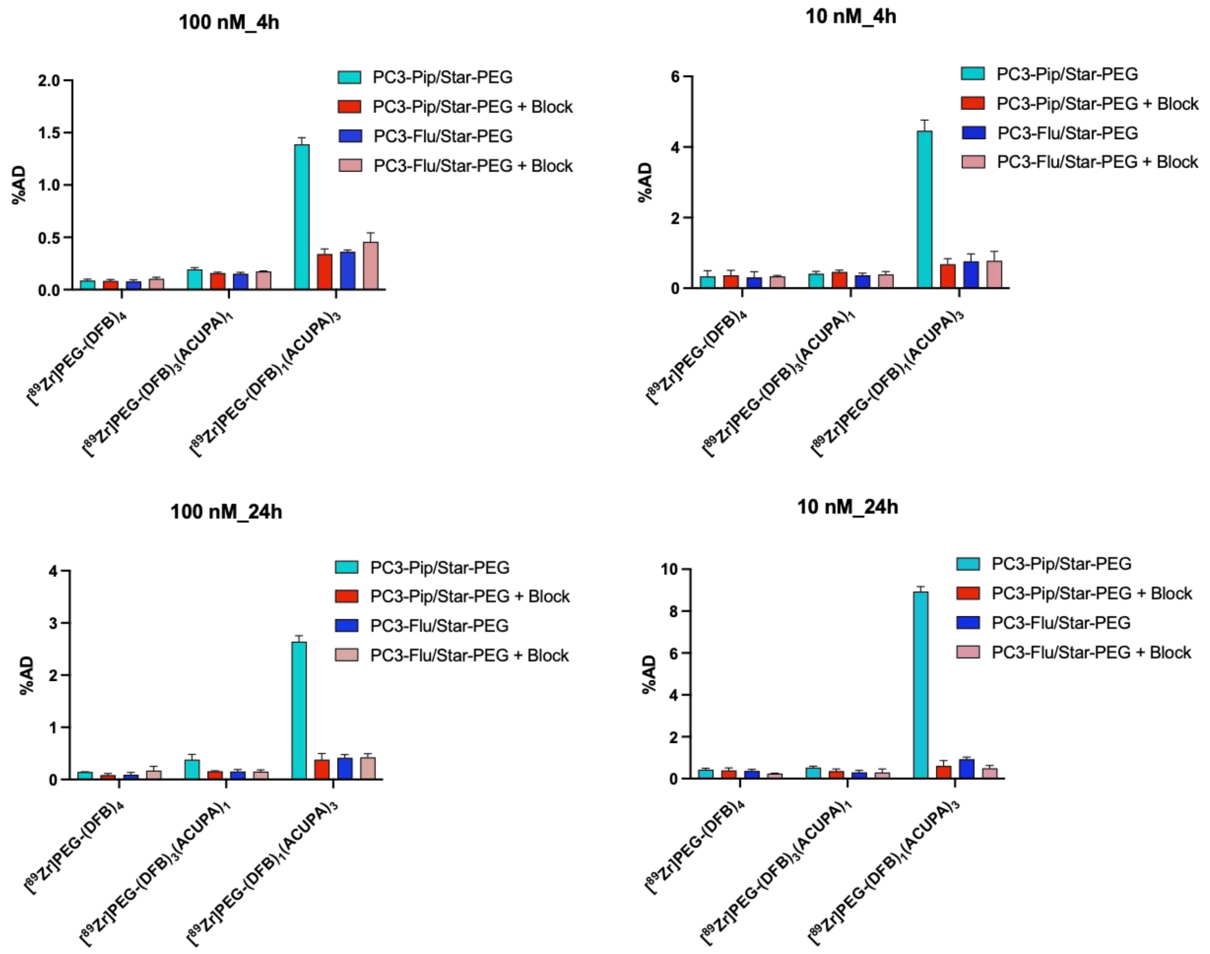

Figure S19. *In vitro* blocking assay of ^{89}Zr labeled nanocarriers at different concentration at 4 h and 24 h incubation in both PSMA+ PC3-Pip and PSMA- PC3-Flu cells by using PSMA-2 as blocking agent.

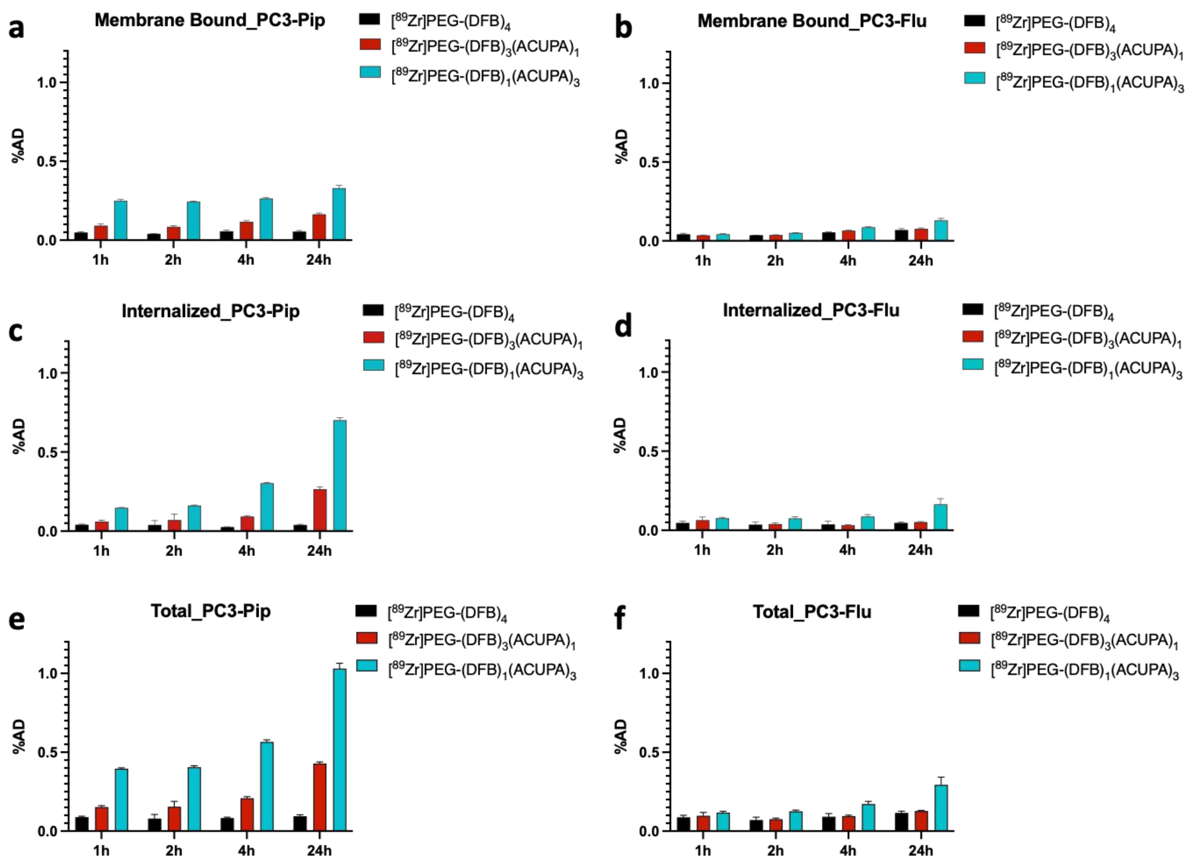

Figure S20. (a,b) Membrane Bound, (c,d) Internalized, and (e,f) Total uptake of the [^{89}Zr]Star-PEGs at 1 μM concentration up to 24 h in PSMA+ PC3-Pip and PSMA- PC3-Flu cells. Membrane bound activity was collected by 5 minute of acid wash with cold mixture of 50 mM glycine and 150 mM NaCl.

7. *In vivo* Studies

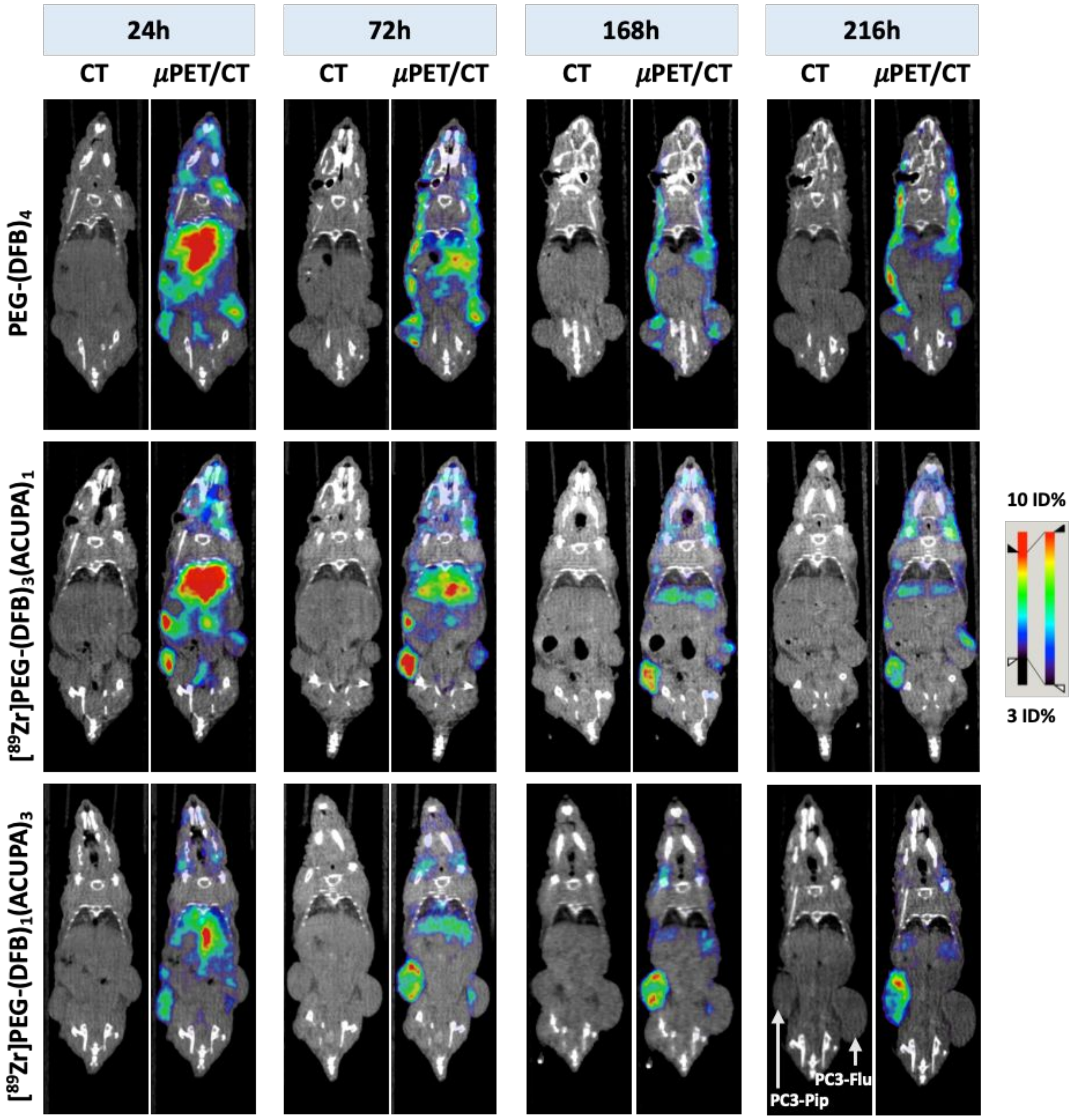

Figure S21. Coronal CT, and coronal μPET/CT fusion images obtained at 24 h, 72 h, 168 h, and 216 h following administration of 89Zr labeled nanocarriers reveal high tumor uptake with low background tissue retention of $[^{89}Zr]PEG\text{-}(DFB)_1(ACUPA)_3$ over time.

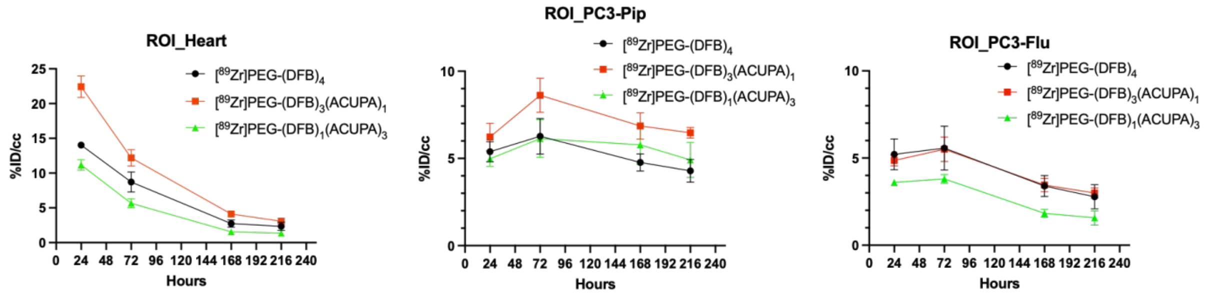

Figure S22. ROI plot on heart, PC3-Pip and PC3-Flu tumors up to 216 h. (n = 4)

Table S4. Region of interest analysis data reported as %ID/g of the ^{89}Zr labeled nanocarriers on heart at 24 h, 72 h, 168 h, and 216 h. (n = 4)

Hours	$[^{89}Zr]PEG-(DFB)_4$				$[^{89}Zr]PEG-(DFB)_3(ACUPA)_1$				$[^{89}Zr]PEG-(DFB)_1(ACUPA)_3$			
24	14.17	13.74	14.17	14.00	22.10	20.79	22.24	24.52	11.67	11.94	10.75	10.35
72	7.11	7.90	9.93	9.95	12.29	11.24	11.43	13.8	6.63	5.25	5.42	5.28
168	2.25	2.34	3.38	2.94	3.59	4.04	4.14	4.67	1.68	1.54	1.42	1.53
216	1.80	1.86	2.94	2.77	2.82	3.01	3.06	3.36	1.46	1.37	1.25	1.35

Table S5. Region of interest analysis data reported as %ID/g of the ^{89}Zr labeled nanocarriers on PC3-Pip tumors at 24 h, 72 h, 168 h, and 216 h. (n = 4)

Hours	$[^{89}Zr]PEG-(DFB)_4$				$[^{89}Zr]PEG-(DFB)_3(ACUPA)_1$				$[^{89}Zr]PEG-(DFB)_1(ACUPA)_3$			
24	5.87	4.97	5.89	4.80	5.46	6.04	7.33	6.05	5.58	4.69	4.63	4.98
72	7.07	5.82	7.15	5.04	7.28	8.62	8.98	9.59	7.64	5.66	5.14	6.09
168	5.32	4.27	5.03	4.43	6.07	6.36	7.45	7.55	7.33	5.08	4.82	5.86
216	4.72	4.18	4.82	3.40	6.14	6.88	6.47	6.39	6.38	4.56	4.15	4.54

Table S6. Region of interest analysis data of the ^{89}Zr labeled nanocarriers reported as %ID/g on PC3-Flu tumors at 24 h, 72 h, 168 h, and 216 h. (n = 4)

Hours	$[^{89}Zr]PEG-(DFB)_4$				$[^{89}Zr]PEG-(DFB)_3(ACUPA)_1$				$[^{89}Zr]PEG-(DFB)_1(ACUPA)_3$			
24	4.841	5.97	5.91	4.13	4.81	4.47	4.93	5.22	3.84	3.47	3.50	3.58
72	4.24	6.89	6.37	4.77	4.73	5.12	6.28	5.88	3.46	3.75	3.93	4.04
168	3.29	4.24	3.24	2.80	3.34	3.69	3.81	2.95	1.87	1.59	1.73	2.12
216	1.97	3.62	2.96	2.54	2.72	3.34	2.79	3.12	1.55	1.11	2.10	1.50

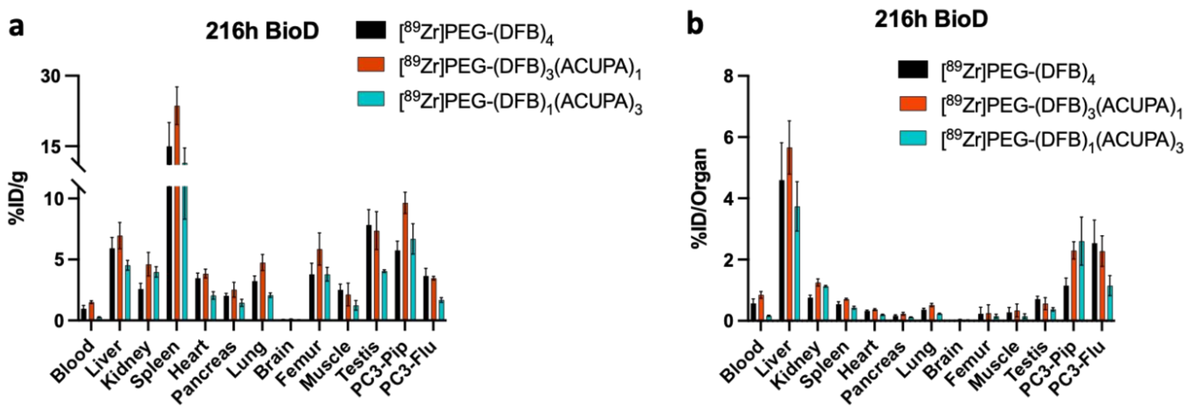

Figure S23. Organ biodistribution presented in (a) %ID/g, and (b) %ID/organ for ^{89}Zr labeled nanocarriers at 216 h postinjection. (n = 4)

Table S7. Organ biodistribution analysis data in %ID/g tissue for ^{89}Zr labeled nanocarriers at 216 h. (n = 4)

**Organs**	**[^{89}Zr]PEG-(DFB)$_4$**				**[^{89}Zr]PEG-(DFB)$_3$(ACUPA)$_1$**				**[^{89}Zr]PEG-(DFB)$_1$(ACUPA)$_3$**			
**Blood**	0.69	0.8	1.16	1.22	1.41	1.67	1.45	1.46	0.32	0.23	0.27	0.24
**Liver**	4.9	5.87	5.87	7.07	6.22	6.51	6.549	8.55	5.04	4.4	4.07	4.58
**Kidney**	2.4	2.41	2.2	3.28	3.96	3.96	4.536	6.01	4.44	3.76	3.5	4.21
**Spleen**	9.9	11.96	16.88	21.18	28.51	23.14	18.745	24.05	16.16	9.85	9.39	10.46
**Heart**	3.06	3.22	3.99	3.63	3.83	3.47	3.732	4.33	2.27	1.84	1.73	2.37
**Pancreas**	1.75	2.08	2.19	2.09	2.22	1.96	2.54	3.37	1.73	1.66	1.16	1.24
**Lung**	2.74	3.01	3.64	3.53	4.19	4.4	4.708	5.7	2.33	1.99	1.91	2.08
**Brain**	0.07	0.08	0.09	0.08	0.1	0.1	0.124	0.1	0.07	0.05	0.06	0.05
**Femur**	4.27	3.07	4.83	3	6.85	4.58	4.878	7.13	4.61	3.43	3.42	3.72
**Muscle**	2.51	2.8	1.85	2.88	1.31	1.9	1.841	3.48	0.83	1.47	0.99	1.67
**Testis**	7.09	7.74	9.65	6.89	6.64	6.6	9.697	6.54	4.15	4.14	3.96	3.96
**PC3-Pip**	6.48	4.99	6.31	5.25	8.41	10.43	9.701	10.03	8.49	6.05	5.72	6.52
**PC3-Flu**	3.02	3.94	4.38	3.29	3.27	3.63	3.462	3.53	1.77	1.4	1.77	1.79

Table S8. Organ biodistribution analysis data in %ID/organ tissue for ^{89}Zr labeled nanocarriers at 216 h. (n = 4)

Organs	[^{89}Zr]PEG-(DFB)$_4$				[^{89}Zr]PEG-(DFB)$_3$(ACUPA)$_1$				[^{89}Zr]PEG-(DFB)$_1$(ACUPA)$_3$			
**Blood**	0.4	0.49	0.7	0.68	0.87	0.97	0.85	0.7	0.18	0.14	0.18	0.15
**Liver**	5.24	3.76	5.99	3.41	6.33	4.69	6.46	5.16	4.15	3.66	4.49	2.64
**Kidney**	0.69	0.77	0.7	0.86	1.1	1.38	1.24	1.29	1.12	1.1	1.17	1.13
**Spleen**	0.44	0.64	0.56	0.53	0.74	0.73	0.69	0.68	0.41	0.46	0.48	0.37
**Heart**	0.3	0.32	0.36	0.32	0.4	0.39	0.34	0.34	0.2	0.17	0.19	0.22
**Pancreas**	0.13	0.19	0.19	0.15	0.2	0.24	0.19	0.29	0.11	0.13	0.13	0.1
**Lung**	0.34	0.35	0.43	0.34	0.47	0.51	0.59	0.5	0.24	0.21	0.25	0.22
**Brain**	0.03	0.03	0.03	0.03	0.04	0.04	0.05	0.03	0.03	0.02	0.02	0.01
**Femur**	0.41	0.05	0.42	0.05	0.66	0.1	0.09	0.16	0.23	0.09	0.17	0.12
**Muscle**	0.11	0.42	0.18	0.4	0.12	0.56	0.2	0.47	0.07	0.19	0.11	0.24
**Testis**	0.64	0.69	0.84	0.68	0.58	0.69	0.7	0.28	0.43	0.32	0.42	0.33
**PC3-Pip**	0.84	1.43	1.12	1.21	2.65	1.98	2.38	2.19	3.73	2.51	1.92	2.26
**PC3-Flu**	2.34	1.71	3.53	2.57	2.07	1.69	2.51	2.83	1.47	1.34	1.05	0.74

References

1. K. Maresca, S. Hillier, F. Femia, D. Keith, C. Barone, J. Joyal, C. Zimmerman, A. Kozikowski, J. Barrett, W. Eckelman and J. Babich, *Journal of Medicinal Chemistry*, 2009, **52**, 347-357.
2. A. Machulkin, Y. Ivanenkov, A. Aladinskaya, M. Veselov, V. Aladinskiy, E. Beloglazkina, V. Koteliansky, A. Shakhbazyan, Y. Sandulenko and A. Majouga, *Journal of Drug Targeting*, 2016, **24**, 679-693.
3. J. Barrett, R. Coleman, S. Goldsmith, S. Vallabhajosula, N. Petry, S. Cho, T. Armor, J. Stubbs, K. Maresca, M. Stabin, J. Joyal, W. Eckelman and J. Babich, *Journal of Nuclear Medicine*, 2013, **54**, 380-387.
